# Exploring the Physicochemical Characteristics of Marine Protein Hydrolysates and the Impact of In Vitro Gastrointestinal Digestion on Their Bioactivity

**DOI:** 10.3390/md22100452

**Published:** 2024-10-01

**Authors:** Deepanshi Sharma, Snehal Gite, Maria G. Tuohy

**Affiliations:** 1 Molecular Glycobiotechnology Group, Biochemistry, School of Biological and Chemical Sciences, University of Galway, H91 TK33 Galway, Ireland; maria.tuohy@universityofgalway.ie; 2 Bio-Marine Ingredients Ireland, Unit 9, Lough Egish Food Park, Co., A75 WR82 Monaghan, Ireland; 3 Ryan Institute and MaREI, SFI Research Centre for Energy, Climate and Marine Research and Innovation, University of Galway, H91 TK33 Galway, Ireland

**Keywords:** fish protein hydrolysates, functional properties, bioactive peptides, amino acids, antioxidant activity, diabetes

## Abstract

Fish protein hydrolysates (FPHs) were obtained from different fish sources using a combination of microbial enzymes. The industrially produced FPHs from blue whiting (*Micromesistius poutassou*) and sprat (*Sprattus sprattus*) were compared to freeze-dried FPHs generated in-house from hake (*Merluccius merluccius*) and mackerel (*Scomber scombrus*) in terms of their physicochemical composition and functionality. Significant differences (*p* < 0.05) were observed in the protein, moisture, and ash contents of the FPHs, with the majority having high levels of protein (73.24–89.31%). Fractions that were more extensively hydrolysed exhibited a high solubility index (74.05–98.99%) at different pHs. Blue whiting protein hydrolysate-B (BWPH-B) had the highest foaming capacity at pH 4 (146.98 ± 4.28%) and foam stability over 5 min (90–100%) at pH 4, 6, and 8. The emulsifying capacity ranged from 61.11–108.90 m^2^/g, while emulsion stability was 37.82–76.99% at 0.5% (*w*/*v*) concentration. In terms of peptide bioactivity, sprat protein hydrolysate (SPH) had the strongest overall reducing power. The highest Cu^2+^ chelating activity was exhibited by hake protein hydrolysate (HPH) and mackerel protein hydrolysate (MPH), with IC_50_ values of 0.66 and 0.78 mg protein/mL, respectively, while blue whiting protein hydrolysate-A (BWPH-A) had the highest activity against Fe^2+^ (IC_50_ = 1.89 mg protein/mL). SPH scavenged DPPH and ABTS radicals best with IC_50_ values of 0.73 and 2.76 mg protein/mL, respectively. All FPHs displayed noteworthy scavenging activity against hydroxyl radicals, with IC_50_ values ranging from 0.48–3.46 mg protein/mL. SPH and MPH showed the highest scavenging potential against superoxide radicals with IC_50_ values of 1.75 and 2.53 mg protein/mL and against hydrogen peroxide with 2.22 and 3.66 mg protein/mL, respectively. While inhibition of α-glucosidase was not observed, the IC_50_ values against α-amylase ranged from 8.81–18.42 mg protein/mL, with SPH displaying the highest activity. The stability of FPHs following simulated gastrointestinal digestion (SGID) showed an irregular trend. Overall, the findings suggest that marine-derived protein hydrolysates may serve as good sources of natural nutraceuticals with antioxidant and antidiabetic properties.

## 1. Introduction

Unwanted fish components such as heads, viscera, bones, skins, and scales are frequently dumped during inspection in the industrial processing of captured fish. Disposal of fish waste is costly both in terms of time and money, and such waste typically gets tossed overboard and back into the ocean, posing a threat to the environment and human health. According to the latest studies, fish waste may be reprocessed into fish protein hydrolysates (FPHs), which have the potential to offer a valuable range of functional and bioactive properties. Positive health benefits may be achieved through the inclusion of FPH in diets as a high-protein additive. Additional benefits of FPHs, as well as enhanced functionality, include a highly soluble and stable product.

Enzymatic hydrolysis is the most effective and reliable method of generating FPHs [1,2,3]. Reaction conditions are gentler in contrast to chemical hydrolysis, offering greater process control and specificity and the generation of end-products (peptide hydrolysates) with reduced likelihood of adverse reactions. Peptides and amino acids (AAs) produced by enzymatic hydrolysis are reported to be bioactive. Their molecular structures suggest that they may have numerous beneficial properties, such as reducing oxidative stress, that improve overall organism health [4]. When developing target-based products, specificity can be ensured by using proteolytic enzymes (proteases or peptidases) with known catalytic properties. Furthermore, the processes used to generate FPHs require careful consideration as these can affect their molecular size, surface charge, and peptide sequences, all of which impact their nutritional composition and utility [3].

The use of FPHs as food ingredients and nutraceuticals is influenced by their overall chemical composition and AA availability. High protein content in FPHs has been reported in several studies (values ranging from 65–95%) [5,6,7], which is probably the result of protein solubilisation during hydrolysis and separation from insoluble substances during fractionation. In addition to their high protein content, a significant number of studies confirm that FPHs have less than 10% fat content [5,6,7,8], which is a favourable attribute for dietary components since it minimises the likelihood of oxidation, which is a concern when dealing with the shelf-life of food products. Liquid FPH is challenging for long-term storage and transportation; hence, dried FPH is preferred for its stability. The higher temperatures used during hydrolysis and drying are known to result in a moisture content of 0–5% in the final product. Generally, the goal of processing is to generate a hydrolysate high in protein with a low moisture content, for which a suitable drying technique is critical.

The functionality of hydrolysates is defined by their physicochemical characteristics, which in turn govern how they perform in food systems during the stages of processing, preservation, preparation, and consumption [9]. In addition, molecular size, conformation, net charge, AA sequence, and composition also affect functionality. Reaction conditions such as pH, temperature, duration, degree of hydrolysis (DH), and enzyme choice can be modified to further enhance the functional properties of proteins through controlled enzymatic hydrolysis. Each of these variables directly affects the protein’s molecular size and ionic groups, and, when optimised, the hydrolysates produced can have outstanding functionality in terms of their ability to retain water and oil and their solubility, foaming, and emulsifying properties. Examining the AA profiles of hydrolysates is critical to understanding their bioactivity and functional properties. For example, the presence of hydrophilic AAs, such as histidine and serine, and understanding their interaction with water can provide insights on the function and structure of hydrolysates.

The potential of bioactive peptides derived from marine by-products as functional and health food ingredients has recently attracted a lot of attention. These substances provide a source of vital minerals and nutrients, in addition to bioactive properties such as antioxidant [10,11], antihypertensive [12], antidiabetic [13,14], and antimicrobial [15]. Short-chain bioactive peptides have enhanced functionality compared to the parent protein due to the generation of new active sites during protein hydrolysis. These sites can bind to molecules in the body (e.g., cell receptors) and exert a physiological change. The most commonly reported bioactivity of FPHs is their antioxidant potential. Free radicals are extremely unstable molecules that can react with other molecules in a biological system and exert oxidative stress, which can damage cell structures. Over time, the cumulative effects of oxidative stress can result in a number of serious illnesses, including cardiovascular diseases [16], cancer [17], and neurodegenerative disorders [18]. Antioxidants, as the name implies, work to reduce oxidative stress by delaying or inhibiting the oxidation of a substrate. Several factors can determine the structure and bioactivity of the FPHs, ranging from the type of fish by-product to the reaction conditions and the type of enzyme used. Based on their size, composition, and sequence, peptides generated may have the ability to scavenge free radicals or reduce the overall antioxidative stress in a system [19]. A few potent synthetic antioxidants have been found that are effective against oxidation by interacting with free radicals and inhibiting the effect they have on a system. However, safer and naturally derived substitutes that can effectively prevent or slow down the oxidation process without causing any adverse effects are needed.

In addition to antioxidant potential, peptides have been reported to have antidiabetic properties. Diabetes mellitus is a high-risk condition in which the body is unable to produce sufficient insulin, resulting in excessive blood glucose levels. Diabetes raises the risk of various additional complications, including heart conditions [20], osteoporosis [21], and sight loss [22]. The prevalence of this disease is steadily rising, and the implementation of drugs that can regulate blood glucose levels is the focal area for treatment. Synthetic medications are available for the treatment of diabetes. However, to alleviate the likelihood of adverse reactions, the focus has shifted to positioning natural alternatives onto the pharmacological market. One of the ways that dietary ingredients may exert an anti-diabetic effect is by targeting the key enzymes involved in balancing the blood glucose levels, such as α-amylase, α/β-glucosidase [5], and dipeptidyl peptidase (DPP-IV) [13]. Bioactive peptides developed from fish waste have been reported to exhibit inhibitory activities against these enzymes, with the combination of unique sequences and AA composition, as well as exposed surface charges, thought to underpin the physiological effect.

In this study, FPHs were generated from different species of fish, specifically, blue whiting (*Micromesistius poutassou*), sprat (*Sprattus sprattus*), hake (*Merluccius merluccius*), and mackerel (*Scomber scombrus*), with the objective of comprehensively characterising their physicochemical features along with exploring their bioactive potential in the form of their antioxidant and antidiabetic capabilities. Furthermore, digestion of the FPHs by enzymes commonly found in the GI tract in an in vitro system was carried out to test for any loss or deterioration in observed bioactivity.

## 2. Results and Discussion

The soluble protein content of test and control samples for hake and mackerel by-products was determined at 0 and 4 h time intervals. The data (not included) indicated that the soluble protein content from hake mince was higher in hydrolysates generated using Flavourzyme. On the other hand, for mackerel, the soluble protein content was higher following hydrolysis with Alcalase. Based on these outcomes, two FPHs with the highest protein content (HPH and MPH) were selected and freeze-dried for further analysis.

### 2.1. Proximate Analysis of FPH

The composition of hydrolysates is influenced by the type of by-products utilised in their production, such as viscera, heads, frames, trims, or a mince of these components, as well as their chemical makeup (protein and fat content). The proximate composition of the FPHs recovered from fish waste is presented in Table 1. Protein, moisture, ash, and lipid contents differed significantly between the FPHs (*p* < 0.05).

Protein content was found to be the highest for BWPH-B (89.31 ± 0.11%) and lowest in SPH (59.34 ± 0.19%). A significant difference (*p* < 0.05) was observed between the two FPHs from blue whiting, BWPH-A and BWPH-B. While the latter is a water-soluble fraction, the former is a partly hydrolysed insoluble fraction, which had a lower protein content. The BWPH-B reported here had a higher protein content compared to other findings [2,23]. Overall, soluble protein levels were comparable to other studies carried out with blue whiting [24,25,26]. The total protein content observed for BWPH-A was comparable to the hydrolysates from blue whiting (70.1–74.7%) generated in a different study [5]. The in-house FPHs, HPH and MPH, had a significant soluble protein content (77.56 ± 0.04 and 74.76 ± 0.1%, respectively). HPH had a better protein yield than hydrolysates generated from undersized hake (68%) in a different investigation [1], but not as high as hydrolysates generated from hake heads (84.75–87.92%) [27]. MPH had similar results to mackerel hydrolysates (77.86–80.58%) produced in one other study [28], but higher yields of 82.4 and 92.1% were reported in two other studies [6,29]. The difference between the findings could be due to compositional variability of the by-product utilised for hydrolysis. Another important consideration is that the studies were conducted with a different type and concentration of enzymes, which will affect hydrolysate variability. As mentioned above, SPH had the lowest soluble protein yield in this study. The soluble protein content in all other FPHs ranged from 73–89%, which suggests their value as essential sources of proteins.

The lipid content in the extensively hydrolysed proteins was very low (0.1–0.8%), which contrasted with the partially hydrolysed products. This observation could be due to better release of lipids during an extended hydrolysis period and post-hydrolysis processing, in combination with the type of fish species/by-product used to prepare the FPH. BWPH-B had the lowest lipid content (0.18 ± 0.00%), which was slightly better than the blue whiting hydrolysates (0.2–0.3%) obtained in a different study [26]. It has been suggested that an optimal FPH should have less than 0.5% of fat content [30]. In this study, only one of our FPHs (BWPH-B) meets that requirement. Although mackerel naturally has a high oil content, MPH (and HPH) was not too far from the suggested ideal (*p* <0.05). A lower lipid content improves the stability of the FPH to oxidation, thus enhancing its shelf life.

Ash content is a measure of the overall inorganic or mineral composition in the hydrolysate. In the current study, the FPHs had an ash content ranging from 7–13%, with the lowest value observed for BWPH-A (7.59 ± 1.18%). The difference between the ash contents of BWPH-A and BWPH-B was significant (*p* < 0.05), but both were lower than the blue whiting hydrolysates (12.6–19.3%) generated in a different investigation [26]. The slightly higher ash content observed in HPH and MPH could be due to the addition of alkali agents to adjust the pH during hydrolysis. In addition, the presence of residual bone in the mince, which is a major source of minerals, could also increase the value. In spite of this, the ash content of MPH was found to be lower than other findings that reported values ranging from 13.16–14.14% [28].

Finally, the carbohydrate content was very low in all FPHs (<1%), which is an important observation, as the presence of free sugars may potentially alter the chemical and functional characteristics of the hydrolysate. A highly soluble protein content combined with low levels of fat, moisture, and ash are positive characteristics for a hydrolysate to meet acceptability criteria as an ingredient for human consumption.

### 2.2. Functional Properties of FPH

#### 2.2.1. Solubility and Turbidity Profiles

**Figure 1:**
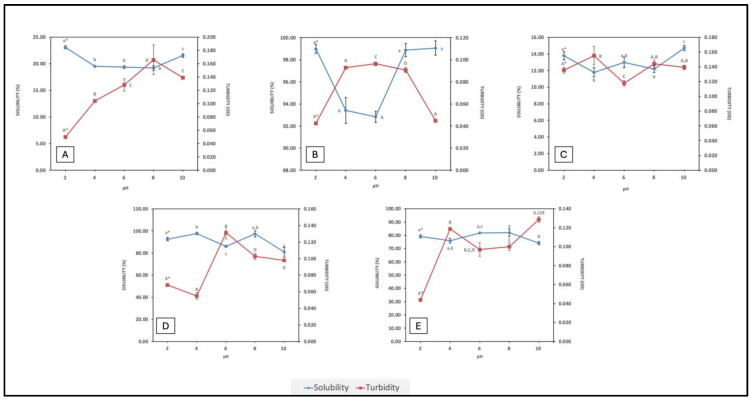
Solubility vs. turbidity profiles of FPHs: (**A**) BWPH-A: blue whiting protein hydrolysate A; (**B**) BWPH-B: blue whiting protein hydrolysate B; (**C**) SPH: sprat protein hydrolysate; (**D**) HPH: hake protein hydrolysate; (**E**) MPH: mackerel protein hydrolysate. Error bars are expressed as mean ± SD (*n* = 3). * Different letters in a category indicate significantly different means (*p* < 0.05).

Solubility is an essential attribute since it directly influences the other functional traits in proteins, such as foaming and emulsifying properties. It is a direct consequence of the extent of hydrolysis and amino acid composition of the peptides formed. Smaller peptides with a higher content of hydrophilic groups are more soluble; exposure of hydrophilic groups facilitates hydrogen bond formation with water molecules. Peptides with a higher content of hydrophobic amino acids, of longer peptides that can form secondary structures, may be less soluble, depending on composition and conformation. In contrast, turbidity is a visual measure of particle aggregation in a solution. There is an inverse correlation between solubility and turbidity. Solubility of FPHs across a wide pH range offers insight on their implementation in food formulations. Isoelectric properties of peptides can influence peptide solubility within a given pH range (i.e., peptides and proteins are least soluble or partially soluble 0.5–1.0 pH unit either side of their isoelectric points (pI)). Associations between peptides in an FPH, due to hydrophobicity, π-π stacking, hydrogen bonding, electrostatic, and van der Waals interactions, can all influence solubility. Solubility and turbidity profiles of the FPHs over a pH range from 2–10 are displayed in Figure 1.

Each hydrolysate followed a relatively unique trend in terms of solubility at different pHs. As expected, the findings revealed that the solubility of BWPH-A was low; this FPH was the insoluble fraction recovered from blue whiting after enzymatic hydrolysis. In contrast, BWPH-B, which was extensively hydrolysed fraction, exhibited excellent solubility across a range of pHs (from 92.85 ± 0.51% at pH 6 to 99.06 ± 0.65% at pH 10). In a different study, solubility of hydrolysates from blue whiting at a DH of 15% was around 70% [26]. Both soluble and insoluble FPHs from blue whiting displayed least solubility around pH 6, which could be due to the pI of peptides present. SPH also exhibited poor solubility (i.e., 11.79 ± 0.56% at pH 4 to 14.71 ± 0.31% at pH 10). Our findings were similar to one particular study, in which blue whiting treated with Alcalase yielded an FPH with a solubility range of 90–100% over a wide pH range [24]. HPH had an excellent solubility profile, which was quite similar to the profile for BWPH-B. Solubility was highest at pH 4 (97.78 ± 1.15%) and lowest at pH 10 (80.87 ± 4.46%). MPH also demonstrated good solubility, with solubility highest at pH 8 (81.98 ± 2.78%) and lowest at pH 10 (74.05 ± 1.22%). To the author’s best knowledge, this is the first study to explore the solubility of protein hydrolysates obtained from mackerel and hake from Atlantic waters across a wide range of pH. The difference in overall solubility between the FPHs could be due to the length and ratio of hydrophobic to hydrophilic AAs in peptides present in the respective FPHs. For the in-house FPHs, an increase in solubility at pH values below and above their probable pIs was observed. Under these conditions, electrostatic repulsion between molecules would be greater than the hydrophobic interactions that promote protein aggregation and precipitation. Overall, the FPHs reported here that displayed good solubility profiles over a wide pH range underpin their potential in food formulations. The FPHs having low solubility (<25%), while more challenging in terms of applications, are nonetheless of interest to see if they display any functional or bioactive properties.

From the graphs, it is evident that pH also plays an important role in the turbidity of a FPH solution. BWPH-B had the highest solubility range and, consequently, the lowest turbidity. It is also interesting to note the relationship between turbidity, solubility, and pH. For example, for HPH, solubility was lowest at pH 6, which was also the pH at which solution turbidity was highest. Similarly, turbidity was high for BWPH-A at pH 6 and 8, where low solubility was observed.

#### 2.2.2. Water and Oil Holding Capacity and Bulk Density

The ability of a food ingredient to retain water is generally a desirable functional trait since it ensures that the food’s texture remains consistent over time [31]. The AA composition of hydrolysates has a significant impact on their water holding capacity (WHC). Naturally, FPHs with exposed hydrophilic AAs can interact more effectively with water, resulting in a greater WHC. However, excessive hydrolysis, which results in increased free AAs, has been shown to reduce WHC [32]. The oil holding capacity (OHC) of a hydrolysate improves palatability in food products and is significantly influenced by a compound’s hydrophobicity. In hydrolysates obtained through denaturation (post-hydrolysis), hydrophobic groups are exposed to the environment, and these hydrolysates have been reported to have superior OHC. The WHC and OHC values of FPHs obtained in this study are presented in Table 2.

The FPHs exhibited a significant difference (*p* < 0.05) between their values for WHC and OHC values. A value of 3.5 g water absorbed per g of FPH was recorded for BWPH-A, which was higher than hydrolysates from bluewing searobin (2.4 g water/g) [33]. On the other hand, BWPH-B had a lower value (1.25 ± 1.06 g/g), which probably correlates with it being more hydrolysed to smaller peptides and free AAs. In contrast, BWPH-A and SPH displayed the highest WHC. The WHC of HPH and MPH were very low (0.15 ± 0.10 and 0.75 ± 0.35 g/g, respectively). The OHC values of all FPHs varied significantly (*p* < 0.05) from 2.53–3.62 g oil per g FPH, which were similar to those reported for Chinese sturgeon FPH (2.59 g oil/g) [34]. Similar to findings for WHC, BWPH-A had the highest OHC (3.62 ± 0.08 g/g), which could be due to partial unfolding of protein and consequent exposure of hydrophobic groups that may promote the OHC properties of the hydrolysate.

Bulk density (BD) is an important characteristic for a food ingredient. In simple terms, it is the ratio of the mass of particles of a substance divided by the volume they occupy. The total volume accounts for the internal particle pore volume, the inter-particle void volume, and the particle volume itself. A high BD means the product is less bulky and occupies less space, making it favourable as a feed. There was a clear difference between the BD of the FPHs produced in this study. The lowest BD (0.60 ± 0.04 g ml^−1^) was recorded for MPH, while BWPH-B had the highest (0.76 ± 0.01 g ml^−1^).

It is interesting to correlate the OHC of the hydrolysates to their bulk density. In powdered additives, the particle density plays an important role in the entrapment of oil. Powders with low bulk density entrap more oil. In this study, BWPH-A and MPH had the lowest BD and highest OHC values in comparison with the other FPHs tested (Table 2). Furthermore, OHC values for blue whiting FPHs in this study were higher than OHC values of 1.36–1.56 g oil/g for blue whiting hydrolysates reported in another study [24]. To the author’s best knowledge, this is the first study that examined the water and oil holding capacities of hydrolysates obtained from sprat (specifically the insoluble fraction), Atlantic mackerel, and Atlantic hake.

#### 2.2.3. Foaming Properties

**Figure 2:**
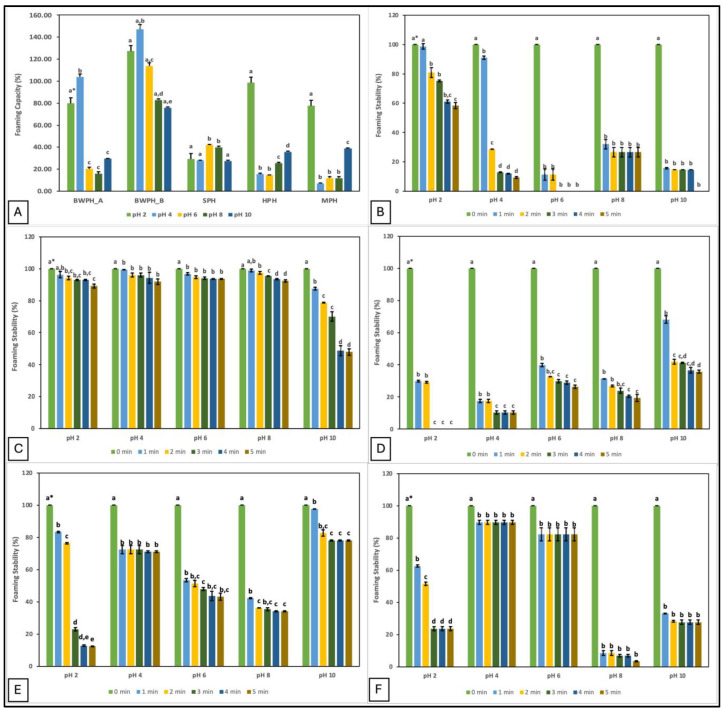
Foaming capacity and stability (%) of FPHs measured over a pH range of 2–10; (**A**) Foaming capacity; (**B**–**F**) Foaming stability (%) of FPHs: BWPH-A: blue whiting protein hydrolysate A; BWPH-B: blue whiting protein hydrolysate B; SPH: sprat protein hydrolysate; HPH: hake protein hydrolysate; MPH: mackerel protein hydrolysate. Error bars are expressed as mean ± SD (*n* = 2). * Different letters in a category indicate significantly different means (*p* < 0.05).

Food additives with good foaming properties contribute to the texture, aroma, and mouthfeel of a product [35]. In the current study, the foaming properties of the FPHs are represented by their foam capacity (FC) and foam stability (FS). FC and FS of FPHs at different pH values (2, 4, 6, 8, and 10) are displayed in Figure 2.

The data demonstrate that pH plays an important role in the foaming properties of protein hydrolysates. The highest FC was observed at pH 2 for HPH and MPH, but at pH 4 for BWPH-A and BWPH-B. This suggests that FC of all FPHs was better at acidic pH, which could in part be due to better solubility. Increased solubility may increase migration of the protein molecules to the air-water interface, thereby lowering surface tension and generating foam. This would explain why the highest values were recorded for BWPH-B at pH 2 (127.21 ± 7.69%) and 4 (146.98 ± 2.49%). In this study, FC for the FPHs was observed to decrease with increasing pH, which could be the result of charge repulsion between the ionic groups of peptides under increasingly alkaline conditions [36]. Poor FC values for SPH and MPH at pH 4 and HPH at pH 6 correlate with the lowest solubility of these FPHs at the respective pHs. Foaming properties are influenced by the AA composition of the hydrolysates. Exposure of hydrophilic and hydrophobic groups enhances peptide/protein orientation at the water and air interfaces, respectively. Increased hydrophobicity improves particle adsorption, resulting in an expanded foam. However, the release of free AAs in the system and protein aggregation may obstruct the interaction between protein and water to give low FC and FS [37].

FS was evaluated by observing the mixture for 5 min and measuring foam volume. Similar to its FC value, BWPH-B had excellent FS (89.24–93.65%) after 5 min at all pHs except pH 10, where FS decreased to 47.86 ± 1.89%. SPH, similar to its FC, did not exhibit a favourable FS profile, ranging from 0–35.76 ± 0.85% across the range of pHs tested. The FS of BWPH-A was almost halved after 5 min at pH 2, whereas in the other conditions (pH 6 and 10), no stable foam was left. FS for the in-house FPHs was maximum at pH 10 and 4 for HPH and MPH, respectively (71.05 ± 0.54 and 89.76 ± 1.23%), which correlated with the lowest points of their solubilities. Protein FS is likely to be lowest in the pH range closest to the pI and improve as pH values further from the pI, at which protein solubility is better. The significant differences (*p* < 0.05) between the individual FPHs investigated here could be an outcome of various factors such as starting sample composition, solubility, protein/peptide conformation, net charge, and charge distribution [38].

#### 2.2.4. Emulsifying Properties

Hydrophilic and hydrophobic groups exposed in hydrolysates work to form and stabilise an emulsion. The hydrophobic groups are drawn to the oil droplets, whereas hydrophilic groups face the aqueous phase, forming a protective layer that keeps the droplets from coalescing. The emulsifying properties of the FPHs in this study were assessed in terms of their emulsifying activity index (EAI) and emulsifying stability index (ESI) at different concentrations (see Table 3).

All FPHs exhibited good emulsifying activity index, with BWPH-B displaying the highest EAI (108.09 ± 3.24 m^2^/g) at a concentration of 0.5% (*w*/*v*). The EAI for HPH was marginally lower at 104.71 ± 19.18 m^2^/g. The high solubility profiles of both FPHs may facilitate the diffusion of particles and enhance the water/oil interaction of ionic groups. The correlation between solubility and EAI was also observed for least soluble FPHs, in that at 0.5% (*w*/*v*), BWPH-A and SPH yielded the lowest EAI values (68.48 ± 0.53 m^2^/g and 61.11 ± 2.13 m^2^/g, respectively). In general, EAI values for the FPHs reported in this study significantly decreased with increasing FPH concentration. This finding suggests that unfolding may be more extensive at lower protein/peptide concentrations, which would increase surface area for formation of an emulsion [9].

In terms of ESI, a direct pattern was observed where the value increased with increasing FPH concentration, suggesting that an increase in protein concentration may allow for a thicker film formation around the oil droplets after homogenisation and prevent flocculation for a longer period, thereby improving the emulsion’s stability. In contrast to the results obtained for EAI, BWPH-B had the lowest ESI (58.77 ± 1.23%) recorded at a concentration of 2% (*w*/*v*). While EAI is associated with solubility, ESI has been suggested to be more dependent on protein composition. Smaller molecular weight peptides are unable to sustain an emulsion once it has formed, forcing it to coalesce [31]. In addition, the presence of more hydrophilic groups does not promote stability, which might be another explanation for the results obtained. Therefore, evaluating the AA composition of the peptides along with gaining an insight into their molecular sizes is important to understand their functionality better.

### 2.3. Amino Acid Composition and Qualitative Analysis

A well-balanced AA diet is critical for growth and survival. Fish proteins are known to have both essential (EAA) and non-essential amino acids (NEAA), which is why they have strong potential as supplements for a balanced diet. FPHs produced post-enzymatic hydrolysis which are composed of short peptide chains consisting of 2–20 AAs and free amino acids (FAA). Studying the composition of AAs in FPHs is important in order to know more about their functional and bioactive characteristics. The concentrations of FAA and total amino acids (TAA) expressed in g/100 g FPH for the FPHs in this study are given in Table 4.

As shown in Table 4, the FAA composition of the FPHs varied significantly, which can be attributed to the type of enzyme used in hydrolysis, the starting fish by-product, and the overall DH. Some EAAs, such as lysine, leucine, and valine, were present in higher amounts than other AAs, and therefore these FPHs may serve as excellent food ingredients. The presence of hydrophobic AAs with alkyl R-groups, such as leucine and alanine, in good amounts ensures enhanced functionality in hydrolysates where hydrophobicity plays a major role, as mentioned earlier. Hydrophilic AAs such as aspartic acid, glutamic acid, serine, asparagine, histidine, and threonine are essential in terms of the water-retaining properties and solubility profiles of hydrolysates. A previous study conducted on pumpkin meal hydrolysates reported a decrease in ∑EAA/∑AA with hydrolysis [39]. In the current study, in agreement with previous findings, the FPHs obtained from more extensive hydrolysis (BWPH-B, HPH, and MPH) had lower ∑EAA/∑AA ratios than the partially hydrolysed FPHs (BWPH-A and SPH). Another study reported a significant loss of AA and reduction in TAA with hydrolysis that generated peptides in the size range of 0.3–0.5 kDa [40], while insoluble and soluble fractions obtained by centrifugation following hydrolysis of Nile tilapia (*Oreochromus niloticus*) revealed that the insoluble fraction had a better AA profile [41].

FAA in the partially hydrolysed FPHs was low when compared to the more extensively hydrolysed FPHs. It is also important to add that FAA are important for flavour enhancement, which is a favourable trait for a food ingredient. Out of all AAs evaluated, glutamic acid and aspartic acid were present in abundance, which was also reported by other studies [42,43]. Aspartic and glutamic acids are multifunctional AAs that help in brain development and hormone regulation in humans [44]. Amongst the EAA, lysine and leucine were present in ample amounts and are known to aid in bone development and protection [45]. Dietary supplementation of these AAs may significantly improve physiological functions in the body.

A common way of evaluating and comparing the AA profile of FPHs is to compute their chemical score, which is an important measure of their nutritional attributes. Chemical scores of the FPHs based on the AA requirements of adult humans and common carp are presented in Table 5. It can be seen that most AAs are present in the FPHs generated in this study in sufficient quantities when compared to the suggested pattern for adult humans, with the exception of methionine and histidine, which make them the limiting AAs. Similar observations were made by other reports, where methionine was the limiting AA in enzymatically obtained FPHs [46,47]. Nonetheless, the AA profiles of the FPHs reported here are acceptable for human requirements. Conversely, the chemical score of all AAs was found to be lower when compared to the suggested requirements for common carp, making all limiting.

Another measure of protein quality is the protein efficiency ratio (PER), which is the unit weight gained by a test animal per unit of protein it consumes. However, for simplification, there are some mathematical equations relevant to the concentration of certain AAs that provide a good estimate of this measure. The calculated PER values along with the equations used are displayed in Table 6. The PER values ranged from 0.71–2.16 for BWPH-A, 1.79–2.06 for BWPH-B, 0.24–1.67 for SPH, 0.69–1.81 for HPH, and 0.55–1.84 for MPH. A PER value of 2.0 or above is generally considered a high-quality protein source, which was shown by both hydrolysates from blue whiting. These results were close to those presented in another study [50], which used Flavourzyme and neutrase to obtain hydrolysates from cod (*Gadus morhua*) waste. The theoretical biological value (BV) of FPHs was also calculated and ranged from 64.27–70.84. It is an estimate of how effectively the EAAs are absorbed by the body through ingestion of food.

### 2.4. Microstructure Characterisation of FPH

**Figure 3:**
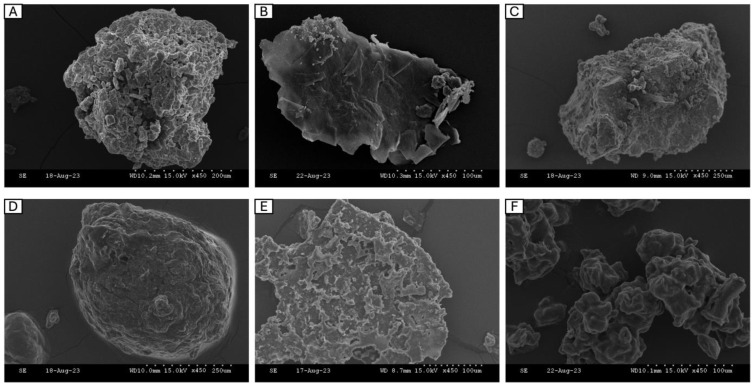
Scanning electron microscopy of FPHs: (**A**) blue whiting protein hydrolysate A (BWPH-A), (**B**) blue whiting protein hydrolysate B (BWPH-B), (**C**) sprat protein hydrolysate (SPH), (**D**) hake protein hydrolysate (HPH), (**E**) mackerel protein hydrolysate (MPH), (**F**) Soy peptone hydrolysate (Soy-PH). Magnification: 450×.

Scanning electron microscopy (SEM) is commonly used to examine the microscopic structure of protein particles in detail following hydrolysis and drying. Studying protein morphology is crucial because it affects its functional characteristics, such as solubility and water retention [51]. SEM images for the dried powders were recorded under similar conditions (magnification: 450×, accelerating voltage: 15.0 kV) and are presented in Figure 3. The structure of the individual FPHs was compared to a commercial hydrolysate obtained from Soy (Soy-PH). The aggregation of particles in the images represents the breakdown of proteins into smaller-sized peptides during hydrolysis. In interpreting the differences between the FPH structure, it is important to note that three of the FPHs, namely BWPH-A, BWPH-B, and SPH, were spray-dried, while HPH and MPH were freeze-dried.

For large-scale FPH production, the drying method commonly employed is spray drying, which uses an atomiser to generate droplets of the liquid solution that are further sprayed and evaporated at high temperature and pressure to produce a solid powder. Due to the application of heat, peptides and proteins may denature, which results in the formation of a more heterogenous surface representative of aggregation. A recent report provided a qualitative review on the morphology of spray-dried particles by examining them on the basis of three types—agglomerate, where individual grains bound together made up a particle; skin-forming, which appears like a non-liquid continuous phase; and finally crystalline, where a large crystal made up a particle [52]. From the images, the insoluble BWPH-A and SPH (Figure 3A,C) looked similar in terms of the presence of round-shaped solid grainy particles that varied in size, indicating that their microstructure type might belong to the group termed ‘agglomerate’. These findings concur with the observation that insoluble or partially insoluble particles tend to take the agglomerate shape [52]. In contrast, BWPH-B (B) appeared crystalline in its particle morphology, which once again agreed with the observation that readily soluble particles tend to be crystalline [52].

Freeze-drying, or lyophilisation, is a much gentler technique that avoids the application of high temperatures, and therefore maintains the structural integrity of proteins. The samples are primarily frozen and thereafter allowed to sublimate under vacuum, yielding solid particles. The particles in freeze-dried FPHs (Figure 3D–F) showed more angularity and appeared bigger in size. Overall, the particles appeared very smooth and glossy in texture, with the presence of pores that may be the result of sublimation of ice crystals formed during freezing. The porous nature of these hydrolysates may also influence their water-holding function.

### 2.5. Structural Characterisation of FPH

**Figure 4:**
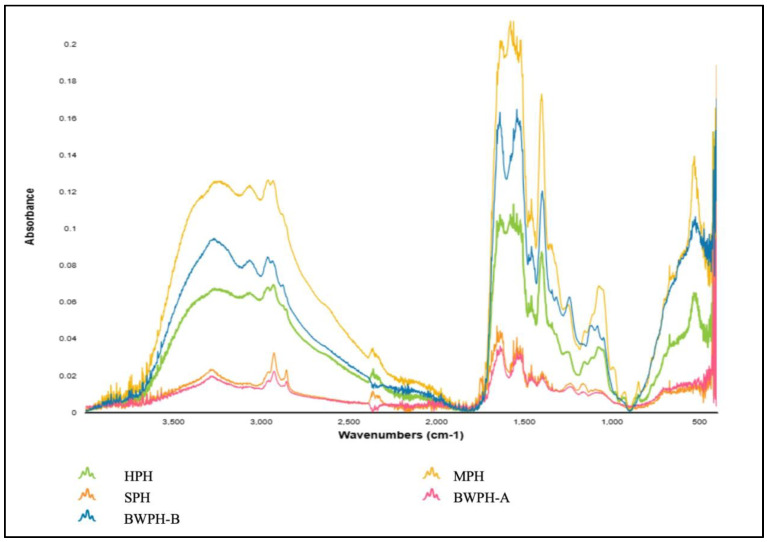
FT-IR spectra produced by FPHs.

Proteins undergo a variety of structural changes during hydrolysis and drying procedures, which can be reflected in the distinctive infrared absorption bands, also known as the amide bands [53]. These changes are caused by the AAs and peptide chains that comprise the secondary structure of protein, which can be detected using fourier transform infrared (FTIR) spectroscopy. The main absorption bands exhibited by proteins in the IR region are namely amide A (3500–3300 cm^−1^), amide I (1800–1600 cm^−1^), amide II (1470–1570 cm^−1^) and amide III (1400–1250 cm^−1^), which help determine their secondary structure. Additional bands include those representing the N-terminal (NH^3+^, ~1510 cm^−1^) and the C-terminal (COO^−^, ~1400 cm^−1^) that provide further information on the structure. Figure 4 illustrates the FT-IR spectra of the FPHs produced in this study.

By examining the spectra, it can be seen that for all FPHs, in amide A, the N-H stretching vibration was detected at a wave number ranging from 3300 to 3200 cm^−1^. As previously stated, this vibration typically occurs between 3500 and 3300 cm^−1^; however, the lower wave number might be the result of hydrogen bonding. The Amide I band occurred between a wave number range of 1700–1600 cm^−1^ for all FPHs, which correlates with C=O stretching vibrations and backbone conformation and is an important indicator for the secondary structure of a protein. The Amide I band for all FPHs except HPH was detected at 1635–1636 cm^−1^, which can be associated with β-sheet formation. HPH had an amide I band at 1653 cm^−1^, which could represent the α-helix present coupled with a glutamine side chain. The amide II band indicates in-plane N-H bending and C-N stretching vibrations, and it spans from 1580 to 1530 cm^−1^. In contrast to amide I, it is less sensitive but provides information on the protein backbone. The absorption peaks for amide III for all FPHs were observed between 1200–1400 cm^−1^, which results from N-H bending and C-N stretching vibrations of the amide bonds. Although amides I and II give stronger signals, amide III can provide distinct details on protein structure without much overlapping of peaks [54]. For instance, an amide III peak for MPH at 1244 cm^−1^ would be characteristic of a β-sheet. The considerable differences between the absorbance values of the FPHs most likely reflect the different starting material, hydrolysis, and drying methods employed.

### 2.6. Degree of Hydrolysis

**Figure 5:**
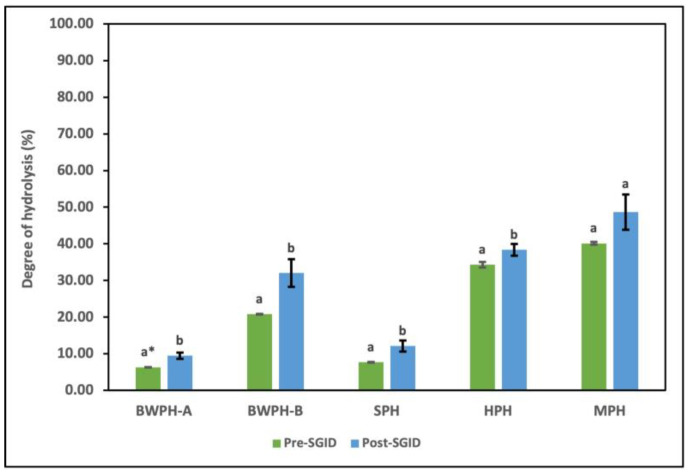
DH (%) of FPHs: BWPH-A: blue whiting protein hydrolysate A; BWPH-B: blue whiting protein hydrolysate B; SPH: sprat protein hydrolysate; HPH: hake protein hydrolysate; MPH: mackerel protein hydrolysate. Error bars are expressed as mean values ± SD (*n* = 3). * Different letters in a category indicate significantly different means (*p* < 0.05).

DH is used as a measure to study the extent of enzymatic hydrolysis in terms of the conversion of proteins into peptides. It offers an insight into the influence of enzyme type and concentration, as well as reaction parameters, on the number of free amino groups generated, which are then compared to the total amino groups in the intact protein. The FPHs that were supplied by Biomarine Ingredients Ireland Ltd. (Monaghan, Ireland), including BWPH-A, BWPH-B, and SPH, were produced under proprietary industrial conditions. Combinations of enzymes such as Alcalase, Flavourzyme, Corolase, neutrase, Endocut-01, and Endocut-02 were utilised for their generation at concentrations ranging from 0.1 to 1%. For the in-house FPHs, Flavourzyme was used to generate HPH, whereas Alcalase was used for the production of MPH, in a reaction time of 4 h. It is also worth revisiting that BWPH-A and SPH are partially hydrolysed insoluble fractions, which has a direct effect on their %DH. The %DH values of each hydrolysate before and after SGID are displayed in Figure 5.

The results for the industrial FPHs corresponded with their partial and extensive hydrolysis profiles, with BWPH-B having the highest %DH (20.82 ± 0.11%). SPH and BWPH-A had the lowest %DH values of 7.69 ± 0.10 and 6.34 + 0.04%, respectively. These results also support the lower concentrations of FAAs detected in BWPH-A and SPH when compared to the other FPHs. The in-house FPHs (HPH and MPH) showed a much higher %DH, which might be attributed to their reaction time. MPH exhibited a greater %DH (40.12 ± 0.38%), which might be due to the enzyme employed (Alcalase) or the nature of substrate. The point of peptide bond cleavage is defined by the enzyme employed; for example, while Alcalase is an endopeptidase with broad specificity, Flavourzyme is a combination of endo- and exopeptidases that cleave peptide bonds at both ends and as well as within a protein. Direct comparison of %DH with functionality and bioactivity of each FPH is not practical since the raw material used is different. The %DH for all FPHs post-SGID were higher than pre-SGID (where SGID represents simulated gastrointestinal digestion). The value for BWPH-B increased to 32.06 ± 3.8%, representing the highest change of all FPHs.

### 2.7. Antioxidant Activity

#### 2.7.1. Reducing Power

A compound’s antioxidant activity is an index of its ability to reduce or inhibit oxidants in a system. This section focusses on using a range of spectrophotometric methods to understand antioxidant capacity. The antioxidant capacity of the FPHs before and after SGID in terms of their reducing power is displayed in Table 7. The activity for FRAP was expressed as µmoles of Trolox equivalents (TE)/g protein. The activities for P-FRAP and PMD were expressed as µmoles Gallic Acid equivalents (GAE)/g protein. The activity for CUPRAC was expressed as µmoles Ascorbic Acid equivalents (AAE)/g protein.

The FRAP assay mainly examines the reducing power of a potential antioxidant by its ability to reduce Fe^3+^ (in the Fe(III)/tripyridyltriazine complex) to Fe^2+^. The ferric reducing ability of a protein indicates its ability to donate electrons or protons for oxidant stabilisation. SPH had the highest antioxidant activity (107.15 ± 4.17 µmoles TE/g protein), followed closely by its GI digested fraction SPH_SGID (100.49 ± 22.15 µmoles TE/g protein). The FRAP activities of the FPHs in this study were in the following order: SPH > MPH > BWPH-B > HPH > BWPH-A. Following SGID, the order changed to: SPH > MPH > HPH > BWPH-A > BWPH-B. The increase in FRAP activity for the partially hydrolysed BWPH-A post digestion could be due to the increased DH (Figure 5), which may expose more reactive groups able to participate in electron donation. Apart from SPH (reduction < 7%), FRAP activity for all other FPHs post digestion increased, which could be linked to a higher number of small peptides and AAs. This suggests that for these FPHs, interaction with GI digestive enzymes would not significantly impair and, in most cases, would enhance their ferric reducing potential. MPH (35.85 ± 0.64 µmoles TE/g protein) had the second-best reducing potential, and SGID substantially enhanced its activity (43.99 ± 2.32 µmoles TE/g protein). Our results were comparable to those published elsewhere [55], where hydrolysed sweet whey with GI digestive enzymes resulted in a FRAP activity of 31.4 ± 1.3 µmoles TE/g protein. SPH, on the other hand, had nearly three times the activity of the aforementioned hydrolysate. Moreover, in comparison to the hydrolysates obtained from the muscle of brownstripe red snapper (*Lutjanus vitta*), where the recorded value was within 10.0 µmoles TE/g protein [56], the FPHs in this study displayed significantly higher FRAP activity. The SPH FRAP activity was comparable to the values observed for the spray-dried hydrolysate from red tilapia (*Oreochromis* spp.) viscera, where activity ranged from 85.0 to 134.7 µmoles TE/g protein, depending on the drying conditions [57].

P-FRAP is a modified FRAP activity assay that determines an antioxidant’s ability to reduce ferricyanide to its ferrous form. FAAs and peptides in the FPHs may serve as electron donors and display substantial reducing power. Consistent with the results obtained with the FRAP assay, the highest P-FRAP activity was noted for SPH (91.67 ± 17.93 µmoles GAE/g protein). However, there was a significant decline (>50%) in activity in post-SGID samples (38.61 ± 6.82 µmoles GAE/g protein), a trend that was similar for all FPHs except BWPH-B. Once again, MPH had the second-best P-FRAP potential (34.30 ± 0.16 µmoles GAE/g protein), closely followed by its GI digested fraction (31.75 ± 2.51 µmoles GAE/g protein), where the reduction in activity was not significant (<8%). Overall, P-FRAP activity values for the FPHs were in the following order: SPH > MPH > BWPH-A > HPH > BWPH-B. Following SGID, the order changed to: SPH > MPH > BWPH-A > BWPH-B > HPH, where the only inconsistency compared to the results obtained with FRAP was the performance of HPH.

The CupRAC (Cupric Reducing Antioxidant Capacity) test determines the total antioxidant capacity of compounds by measuring their reducing power, in this instance—Cu (II) to Cu (I). The CupRAC activity for FPHs produced in this study was in the following sequence before digestion: SPH > MPH > BWPH-A > BWPH-B > HPH, which changed to SPH > MPH > HPH > BWPH-B after digestion. All FPHs yielded a reduction in activity following GI digestion except HPH; for BWPH-A and MPH, the drop recorded was less than 20%, but for BWPH-B and SPH, it was 30 and 50%, respectively. A 30% increase in activity post-GI digestion was noted for HPH, which suggests that its interaction with digestive enzymes would enhance the CupRAC performance of this FPH, perhaps due to the increased DH. The highest activity recorded once again was for SPH (293.18 ± 53.54 µmoles AAE/g protein), followed by its digested fraction (173.47 ± 43.64 µmoles AAE/g protein).

The Phosphomolybdenum assay examines the total antioxidant capacity (TAC) of an analyte based on its ability to reduce Mo (VI) to Mo (V). In this study, all FPHs demonstrated an excellent TAC, with SPH displaying the highest activity (501.74 ± 3.90 µmoles GAE/g protein), followed by its GI digested fraction (269.74 ± 48.44 µmoles GAE/g protein). Although SGID results in a 50% decrease approximately in activity, good TAC levels are retained. A similar trend was observed for two other FPHs, with exceptions being BWPH-B and HPH, where the decrease in TAC was less than 5% post-SGID. The TAC of BWPH-B increased almost two-fold post digestion, which could be due to exposure of hidden polar and non-polar groups following digestion (reflected in the increase in %DH). The increase in TAC for HPH post digestion was low (~5%).

The higher FRAP activity of MPH in comparison to HPH might be ascribed to the utilisation of Alcalase as the digesting enzyme in the former. Alcalase, a broad-spectrum endoprotease, has been reported to produce peptides that have higher antioxidant properties [58,59]. Its cleavage site is located in the middle of a polypeptide chain, targeting peptide bonds on the carboxyl side of Glu, Met, Phe, Tyr, and Lys [60]. Optimal hydrolysis can be attained by using Alcalase independently or in combination with other enzymes [61,62]. Alcalase is also commonly employed to extract peptides rich in hydrophobic AAs, whereas Flavourzyme favours the production of hydrophilic peptides [63]. The suggested antioxidant mechanism for hydrophobic AAs is facilitated by their entry into target areas via hydrophobic interactions with lipid bilayers. Overall, the use of Alcalase alone (in the case of MPH) and in combination with other proteases in the industrially developed FPHs investigated in the current study resulted in greater antioxidant activity (FRAP) in comparison to the FPH obtained using Flavourzyme treatment under the reaction conditions used. In addition to the type of enzyme used, the extent and duration of hydrolysis have an important bearing on the size, sequence, and conformation of the peptides generated, all of which influence final FPH performance. Here, the partially hydrolysed SPH demonstrated much greater reducing power than the other FPHs. BWPH-B, HPH, and MPH had higher AA contents, yet their reducing power was lower than that of SPH, which may be linked to the conformation of peptides following digestion, as suggested by others [64]. Extended hydrolysis might also reduce protein surface hydrophobicity by breaking down hydrophobic regions, thus interfering with their antioxidant capacity. Another possible explanation is that greater quantities of exposed hydrophobic groups on the protein surface may promote protein aggregation and reduce antioxidant capacity [65]. Our findings are consistent with those reported previously [66], where increasing the %DH reduced the reducing power of FPHs. In contrast, another report argued that DH had no link with antioxidant activity and emphasised the importance of the intrinsic properties of the peptides formed [67].

#### 2.7.2. Chelating Activity

Copper and iron are vital heavy metals that play important roles in body function and metabolism, but their accumulation may cause adverse effects, notably the generation of reactive oxidative species (ROS) via Fenton and Haber-Weiss reactions. Compounds that act as metal chelators, thereby preventing the formation of ROS are regarded as effective antioxidants. The purpose of this study is to investigate if the FPHs in this study contain functional groups that can chelate transition metals such as copper and iron and consequently have the potential to block the formation of free radicals. Table 8 displays the iron and copper chelating activities of the FPHs before and after SGID. The findings are reported as half maximal inhibitory concentration (IC_50_) in mg/mL protein concentration. A lower value suggests a greater chelating ability.

In terms of Cu^2+^ chelating activity, all FPHs had noteworthy IC_50_ values, with HPH and MPH having the highest values, pre- and post-digestion. The results indicated that the in-house FPHs outperformed the industrial FPHs, with an IC_50_ value of less than 1 mg protein/mL. In the case of the industrially processed FPHs, the copper chelating activity of SPH (IC_50_ = 1.60 ± 0.09 mg/mL) was better than either of the blue whiting FPHs, and that value only marginally decreased to 1.75 ± 0.09 mg/mL post interaction with digestive enzymes. After GI digestion, both soluble and insoluble fractions of blue whiting showed an increase in chelating activity (~75 and 25%, respectively), indicating that their potency in the digestive tract might not be impacted by the enzymes present and digestion may even improve chelating potential. However, the chelating ability of SPH and HPH decreased (by approx. 16% for both). One probable reason could be the digestive enzymes altering the protein structure, making more ligand-binding atoms available for chelation. S, N, and O atoms can act as ligands in the form of chemical groups such as -SH, -S-S, -NH_2_, =NH, -OH, -OPO_3_H, or >C=O [68]. AAs such as cysteine and methionine contain sulphur atoms, whereas histidine has nitrogen atoms in its aromatic imidazole ring, all of which are known to be efficient copper chelators [69]. Furthermore, N-containing AAs such as glutamine and asparagine and O-containing AAs such as glutamate and aspartate can form complexes with copper ions. As shown, the FPHs in this study contain good amounts of a number of these AAs. The in-house FPHs containing His and Met/Cys had higher FAA concentrations (~ 2 g and 1.25 g/100 g protein, respectively) compared to the other FPHs, which might contribute to their observed higher activity. In addition, they contain higher levels of free glutamine and asparagine, which may be the result of their high %DH, which results in increased FAAs. The FPHs in this study had better chelating ability than FPHs prepared from various fish discards (blue whiting, megrim, red scorpionfish, mackerel, etc.) in another study [5]. In this study, the best IC_50_ value recorded was 2.49 ± 0.02 mg/mL for an FPH prepared from the heads of Atlantic horse mackerel. However, results for the in-house FPHs were more comparable, although fractionally lower, than those reported by [70], where the maximum IC_50_ value for cape hydrolysates was found to be 0.6 mg/mL. In summary, the FPHs showed very promising Cu^2+^ chelating properties, and their interaction in vitro with digestive tract enzymes had little impact on their bioactivity.

As can be seen in Table 8, the Fe^2+^ chelating properties of the FPHs were not as promising as those for Cu^2+^ chelation, with the best and worst IC_50_ values of 1.89 ± 0.24 and 46.34 ± 0.84 mg/mL recorded for BWPH-A and BWPH-B, respectively. Other noteworthy results were observed for SPH (IC_50_ = 5.18 ± 1.85 mg/mL) and HPH (IC_50_ = 4.67 ± 0.33 mg/mL). During SGID, the Fe^2+^ chelating activity of all FPHs decreased significantly, except for BWPH-B, for which an improved IC_50_ value of 27.18 ± 0.93 mg/mL was determined.

An explanation for the superior Fe^2+^ activity of BWPH-A correlated with its TAA concentration of Met/Cys and Thr (0.19 and 3.17 g/100 g protein), particularly the latter, which was more abundant than for any of the other FPHs. These AAs have been the most frequently proposed for iron chelation. However, the observed reduction in activity following SGID can be explained by a shift in pH conditions in the system, resulting in the lack of stability of the iron-AA complex [71]. Aspartate is another NEAA that is deemed important for iron chelation. Hydroxylation of aspartate by dioxygenases yields α-hydroxycarboxylate, which belongs to a known group of iron metal chelating compounds. The total aspartic acid concentration in BWPH-A (9.5 g/100 g protein) was higher than in the four other FPHs, which would also underpin its remarkable Fe^2+^ chelating potential. The lower Fe^2+^ chelating ability of the in-house FPHs in comparison to Cu^2+^ chelation properties has been observed by other authors. While a 68% increase in Cu^2+^ chelating activity of maize zein was noted after hydrolysis, the Fe^2+^ chelating activity was merely 7% [72]. The maximum Fe^2+^ chelating activity measured in lionfish (*Pterois volitans* L.) muscle protein hydrolysates was 56.33%, compared to 90.98% for Cu^2+^ chelating activity [73]. The results suggest that functional groups in peptides may have different affinities for Cu^2+^ and Fe^2+^ ions, resulting in the observed differences. The IC_50_ values for Fe^2+^ chelation for all FPHs in this study, except BWPH-B, were similar to those reported elsewhere for FPHs Klunzinger’s mullet (*Liza klunzingeri)* muscle, which ranged from 2.12 to 12.16 mg/mL [74]. Results for the strongest performing hydrolysate, BWPH-A in our study, were roughly comparable to those published previously [75], for FPHs generated by post-enzymatic hydrolysis of anchovy (*Engraulis japonicus*) muscle protein. For the latter FPHs, IC_50_ values in the range of 0.13–2.72 mg/mL were obtained, depending on the kind of enzyme employed.

#### 2.7.3. Radical Scavenging Activity

Another type of oxidation-limiting mechanism that antioxidants can display is their free radical scavenging potential. The presence of a lone electron makes them highly unstable in nature, and they typically attempt to extract electrons for pairing from other molecules, resulting in injury to proteins and DNA. The radical scavenging activity of FPHs before and after SGID is presented in Table 9. The values are expressed as IC_50_ in mg/mL protein concentration, with a lower value suggesting a greater scavenging affinity.

2,2-diphenyl-1-picrylhydrazyl (DPPH•) is a stable free radical molecule, and the extent of its quenching provides a measure of a compound’s antioxidant activity. All FPHs in this study were able to scavenge the DPPH free radical, both before and after SGID, with the highest IC_50_ value expressed by SPH and MPH at 0.73 ± 0.11 and 1.74 ± 0.04 mg/mL, respectively, and the lowest by BWPH-B at 22.82 ± 1.10 mg/mL. This finding highlights the potential of the FPHs to contribute a hydrogen atom to the free radical, ultimately stabilising the radical and preventing oxidative stress. BWPH-A also showed promising DPPH scavenging potential (IC_50_ = 2.45 ± 0.17 mg/mL), which was much higher than the corresponding soluble, extensively hydrolysed fraction, BWPH-B. This result, along with the one obtained for SPH, supports the theory that partly hydrolysed proteins with a lower %DH exhibit a higher DPPH scavenging potential, as reported by other authors [76,77]. According to a number of reports, scavenging activity increases up to a certain DH, after which it declines [78,79]. The partial unfolding of proteins, which exposes hydrophobic groups that were previously buried inside the structure, is what is regarded as mediating the initial increase in scavenging activity. Hydrophobic AAs and peptides that have recently been exposed can actively scavenge the DPPH radical due to the greater affinity of radicals for hydrophobic residues. However, when the protein is hydrolysed extensively through prolonged hydrolysis, polar hydrophilic groups become more accessible, increasing the solubility of the hydrolysate but decreasing its overall DPPH radical scavenging activity [80]. That said, the in-house FPHs with a high %DH still yielded favourable results, which proves ultimately that the type of substrate and protease utilised, along with the reaction conditions, greatly influence the type of peptides generated and their consequent antioxidant activity.

Aromatic AAs such as Phe, Tyr, and Trp have been reported to act as good scavengers of the DPPH radical since they can donate a neutralising hydrogen atom [81]. In this study, BWPH-A had the highest TAA content of phenylalanine and tyrosine, followed by MPH and SPH. The same FPHs had the highest concentration of isoleucine, an aliphatic hydrophobic AA reported to be involved in DPPH radical quenching [81]. Tryptophan was not detected in the industrially processed FPHs during AA analysis. However, the in-house FPHs had concentrations of 2.33 and 2.77 g Trp/100 g protein for HPH and MPH, respectively, which may have been due to the milder conditions employed for their production but also could be due to the fish-waste composition. The Phe, Tyr, and Trp content is likely to contribute to the very good scavenging activity of MPH.

The findings of our investigation were in line with those reported in earlier investigations. Following digestion of golden grey mullet (*Liza aurata*) with a selection of enzymes, IC_50_ values in the range of 3.80–5.31 mg/mL were determined for the FPHs produced. Some of the current FPHs displayed IC_50_ values similar to these figures [82]. Papain hydrolysis of Klunzinger’s mullet (*Liza klunzingeri*) muscle yielded an FPH that displayed effective DPPH scavenging (IC_50_ = 2.08–3.18 mg/mL), which is in line with our observations for BWPH-A, SPH, and MPH both before and after GI digestion [74]. Similar values were obtained for FPHs from Chinese sturgeon (*Acipenser sinensis*), prepared using papain and Alcalase (IC_50_ = 3.64 and 3.15 mg/mL, respectively) [59]. However, the hydrolysates produced from the by-products of Cape hake (*Merluccius capensis*) exhibited low DPPH radical scavenging activity, for which an IC_50_ value could not be determined [70]. This difference between results for FPHs from Cape hake [70] and Atlantic hake (our study) is very interesting and potentially highlights differences in processing, enzymes used, and/or reaction conditions used in FPH production.

A second radical scavenging activity assay utilised the radical cation ABTS•+ (2,2′-azino-bis (3-ethylbenzothiazoline-6-sulfonic acid)) to examine the total antioxidant potential of the FPHs. The greatest influence on the ABTS radical cation scavenging activity was observed following GI digestion of the FPHs. Before SGID, the most potent FPHs were SPH and MPH, with IC_50_ values of 2.76 ± 0.05 and 4.13 ± 0.12 mg/mL, respectively. The scavenging activity of SPH decreased slightly (less than 10%) after SGID, while the activity of MPH exhibited a 5% increase.

With an IC_50_ value of 11.17 ± 0.27 mg/mL, HPH exhibited the next most favourable activity, which decreased to 9.96 ± 0.19 mg/mL following SGID. The scavenging activity of BWPH-A and BWPH-B was extremely low. However, the activity of their respective SGID fractions showed a significant increase of 70 and 95%, respectively, with IC_50_ values below 10 mg protein/mL. The gastric digestive proteases used for in vitro analysis were pancreatin and pepsin, both of which are endopeptidases that can break down internal peptide bonds within protein sequences. Pepsin typically cleaves peptide bonds at the carboxylic side of aromatic AAs such as phenylalanine and tyrosine, as well as other aliphatic AAs such as leucine [83]. The antioxidant capacities of synthesised tripeptides with various side chain groups were investigated, and it was discovered that aromatic AAs were strongly associated with ABTS scavenging activity [84]. Therefore, improvement in scavenging activity following SGID could be due to the higher amount of free aromatic AAs in the FPHs investigated in this study that, owing to their structure, can donate a proton to the free radicals from their phenolic or indole group. It is also possible that the enhanced performance by the in-house FPHs stems from the free tryptophan that was detected, as mentioned earlier. Moreover, multiple studies have demonstrated that among a variety of enzymes used to hydrolyse protein sources, pepsin stood out as the most effective for producing a hydrolysate with the highest ABTS scavenging activity. According to researchers, although the cooperative action of pepsin and pancreatin produced hydrolysates from the red macroalgae (*Porphyra yezoensis*) with the highest protein concentration, the hydrolysate generated exclusively by pepsin demonstrated the highest ABTS scavenging activity [85]. An assortment of enzymes was used to hydrolyse yellowfin sole (*Limanda aspera*), including Alcalase, a-chymotrypsin, papain, pepsin, pronase E, neutrase, and trypsin, and despite having the lowest DH, the pepsin hydrolysate had the greatest antioxidative action [86]. These results indicate that the type of enzyme and their mode of action may impact the antioxidant capabilities of hydrolysates. The IC_50_ values for MPH both before and after SGID were only marginally better than those reported elsewhere for an FPH from Atlantic horse mackerel (4.56–4.93 mg/mL) [5]. However, in this same study, the performance of hydrolysates from blue whiting surpassed the blue whiting FPHs in this study, which suggests that the type of by-product (substrate) in combination with the enzyme applied can influence bioactivity [5]. The hydrolysate obtained from hybrid sturgeon (*Huso dauricus* × *Acipenser* schrenckii) following bromelain treatment had an IC_50_ value of 3.81 mg/mL against the ABTS radical, which was better than most FPHs in this study, with the exception of SPH/SPH_SGID [87].

The hydroxyl radical (•OH) is one of the core stressors for oxidative damage because of its high non-specific reactivity with compounds [88]. Hydroxyl radical formation is facilitated via the Haber-Weiss and Fenton reactions, wherein the ferrous ions formed during the Haber-Weiss reaction react with hydrogen peroxide in the Fenton reaction, resulting in ROS. In this study, the hydroxyl radical scavenging potential of FPHs was evaluated. Strong scavenging activity was observed in all FPHs, with SPH, MPH, and HPH displaying the highest IC_50_ values at 0.49 ± 0.27, 0.62 ± 0.02, and 0.66 ± 0.16 mg/mL, respectively. The scavenging potential of both in-house FPHs (HPH and MPH) decreased slightly after SGID (<10%; see Table 9), but for SPH, the value decreased by half to 0.89 ± 0.10 mg/mL. Pre-digestion, BWPH-A exhibited an IC_50_ value of 1.10 ± 0.21 mg/mL; this value improved to 0.84 ± 0.14 mg/mL following SGID. Overall, SGID-treated BWPH-B demonstrated the highest scavenging capacity, with an IC_50_ value of 0.48 ± 0.14 mg/mL, which was a notable improvement over the pre-SGID activity. The observed increase, and in some cases stability in scavenging activity for almost all FPHs following SGID, suggests that the bioactivity of peptides following interaction with digestive enzymes such as pepsin and pancreatin in the gastric system would remain largely unaffected. Moreover, based on the mechanism of the Fenton reaction, a parallel can be drawn between the iron (Fe^2+^) chelating ability of FPHs and their hydroxyl radical scavenging potential. Excluding BWPH-B, all FPHs had a chelating potential ranging between 1.81–12.09 mg/mL, which correlates well with hydroxyl radical scavenging potential. Several studies in the literature have reported an increase in hydroxyl radical scavenging activity of proteins post-interaction with digestive enzymes. For example, in vitro digests of buckwheat protein increased in •OH scavenging activity post digestion with pepsin and pancreatin [89]. Similarly, an increase in radical scavenging activity following SGID of different fish soups was observed and was attributed to the presence of peptides having effective hydrogen or electron donors to capture the hydroxyl radical [90]. On the other hand, another study observed no clear trend between the scavenging effect and SGID of protein hydrolysates prepared from Cape hake (*Merluccius capensis*) by-products [70].

The preceding sections have highlighted the potential significance of aromatic AAs in the scavenging power of peptides. This concept is supported by the present investigation, in which higher hydroxyl radical scavenging activity was noted for the FPHs with a higher level of Phe + Tyr, as well as Trp in the in-house FPHs. Almost half of the TAA in the FPHs in this study is comprised of hydrophobic AAs, which most likely underpins the scavenging of free radicals. Based on their physicochemical properties, hydrophobic AAs are known to serve as suitable hydroxyl radical scavengers due to their ability to form hydrophobic interactions with lipid bilayer membranes. According to another study [91], the hydrolysate with the highest concentration of hydrophobic AAs from silver carp (*Hypophthalmichthys molitrix*) exhibited the greatest capacity for scavenging hydroxyl radicals. Almost half of the TAA in present FPHs is made up of hydrophobic ones, thus facilitating the scavenging of free radicals. Our findings were similar to a study that reported an IC_50_ value of 0.74 mg/mL for enzymatically generated hydrolysates of tilapia (*Oreochromis niloticus*) skin gelatin [92]. Squid (*Todarodes pacificus*) hydrolysate yielded an IC_50_ value of 3.4 mg/mL, which was much similar to BWPH-B but significantly higher than other FPHs in our study [93]. An FPH from papain-treated grass carp (*Ctenopharyngodon idellus*) was able to scavenge 50% of hydroxyl radicals at a concentration of 8.12 mg/mL, which was significantly higher than FPHs tested in the current study [94].

Another ROS that plays an active part in cellular stress is the superoxide radical (O_2_•−), a highly reactive anionic compound formed as a result of an oxygen molecule acquiring an electron. While the superoxide radical scavenging activity was lower than that of activity against •OH, a few noteworthy results were obtained for SPH, MPH, and HPH in this study, with IC_50_ values of 1.75 ± 0.16, 2.53 ± 0.13, and 3.18 ± 0.22 mg/mL obtained, respectively. A recurring trend for all FPHs was the decline in scavenging activity following SGID. Other researchers performed simulated digestion on cocoa shell through four phases—oral, gastric, intestinal, and colonic [95]. The superoxide radical scavenging potential of the extract was at its highest at the initial oral phase and decreased by 60.4% during the next three phases. Loss of activity was attributed to the possible destruction of scavenging peptides. In this study, loss of activity following SGID contrasts with the results of hydroxyl radical scavenging and suggests that a FPH’s antioxidant ability may be more significantly impacted by its peptide sequence. The superoxide scavenging activity of blue whiting fractions was found to be the lowest among all the FPHs tested in this study. BWPH-A and BWPH-B had IC_50_ values of 5.69 ± 0.53 and 6.66 ± 0.75 mg/mL, respectively, which decreased by about 50 and 70%, respectively, post SGID. Our findings are comparable to those published by other studies—grey mullet (*Mugil cephalus*) protein hydrolysates had an IC_50_ value of 1.294 mg/mL against superoxide anion radical, which was quite similar to SPH [96]. Another study on peptides prepared from Oyster (*Ostreaplicatula gmelin*) gave an IC_50_ value of 7.02 ± 0.48 mg/mL [97], which was higher than all FPHs in this study before SGID but lower than the corresponding SGID fractions.

The consistent scavenging abilities of the best performing FPHs, SPH, HPH, and MPH are probably due to their AA composition. The in-house FPHs have the highest concentration of free aromatic AAs and, with SPH, the highest content of total Tyr + Phe and thus may facilitate better electron donation due to their phenolic structure. Furthermore, the presence of AAs such as His, Cys, Pro, and Ala are also reported to have high scavenging activity, [98]. All of these AAs are present in high amounts in the in-house FPHs.

The final type of ROS that the FPHs were tested against was hydrogen peroxide (H_2_O_2_). Although H_2_O_2_ is not a reactive substance by itself, it may initiate the Fenton reaction, which produces other harmful free radicals such as •OH. According to the results, all FPHs showed peroxide scavenging activity; however, values were not as notable as their hydroxyl or superoxide radical scavenging potential. The highest H_2_O_2_ scavenging activity was displayed once again by SPH (IC_50_ = 2.22 ± 0.40 mg/mL) and MPH (IC_50_ = 3.66 ± 0.13 mg/mL) and decreased significantly after SGID. This trend was common in all FPHs except for BWPH-B, for which a 25% increase in its IC_50_ value was observed. These results suggest that it is likely that extended hydrolysis of our FPHs (except for BWPH-B) could have resulted in the loss of peptides that before SGID had strong scavenging power and influenced activity more than the DH achieved in FPH preparation. A similar observation was made for gelatin hydrolysates from unicorn leatherjacket (*Aluterus monoceros*) skin, which were prepared using partially purified glycyl endopeptidase (GE) from papaya latex [99]. After further hydrolysis by the enzyme, the H_2_O_2_ scavenging activity decreased.

The concentrations of hydrophobic AAs in the FPHs in the current study were found to be very similar to that of squid (*Todarodes pacificus*) hydrolysate [93]. However, the corresponding IC_50_ value for H_2_O_2_ scavenging (0.1 mg/mL) was much lower than for our FPHs. In another study, hydrolysates from the solitary tunicate (*Styela clava*) had IC_50_ values in the range of 0.9–2.25 mg/mL, which was comparable to SPH from the current study [100].

#### 2.7.4. Lipid Peroxidation Inhibition Activity

Oxidative damage tends to affect lipids, resulting in structural rearrangement, the onset and propagation of a chain reaction, and the generation of lipid peroxyl radicals, which are toxic to the body [101]. Antioxidants may donate a hydrogen atom to the peroxyl radical causing its reduction into a stable non-radical compound, and thereby offer protection by terminating the chain reaction. This subsection investigates the in vitro lipid peroxidation inhibition property of FPHs in this study. The lipid peroxidation inhibition activity of FPHs before and after SGID is summarised in Table 10. The values are expressed as IC_50_ in mg/mL protein concentration, with a lower value suggesting a greater inhibition potential.

The FPHs displayed different inhibition values before and after SGID, with only two displaying higher activity after SGID, BWPH-B and HPH (IC_50_ values of 1.66 ± 0.12 and 1.66 ± 0.03 mg/mL, respectively). The pre-SGID HPH also showed an effective performance (IC_50_ = 2.66 ± 0.07 mg/mL), while the activity of BWPH-B post-SGID was nearly 90% higher than for its pre-digested counterpart. This finding suggests that the exposure of BWPH-B to GI enzymes could significantly improve its performance in the gut. By contrast, BWPH-A lost around 75% of its activity after SGID, despite performing strongly before digestion (IC_50_ = 2.25 ± 0.27); potential inhibitory peptides may have been degraded during SGID. Similarly, the positive activity of SPH prior to SGID (IC_50_ = 2.38 ± 0.74 mg/mL) was reduced following SGID (IC_50_, 5.80 ± 0.65 mg/mL). MPH, although demonstrating impressive efficacy in terms of free radical scavenging, did not yield good activity in terms of anti-lipid peroxidation. Its IC_50_ values before and after digestion were 9.18 ± 2.87 and 11.68 ± 1.31 mg/mL, respectively.

Many theories have been proposed for explaining the antagonistic action of bioactive peptides, with most attributed to modifications made to their size, sequence, and structure following hydrolysis. As discussed in previous sections, the peptides may chelate Fe^2+^ ions to prevent subsequent free radical formation. In order to shield lipid molecules from oxidation, the peptides may also create a protective barrier, influenced potentially by aromatic AA content. Furthermore, a chain reaction propagating oxidative effects could be interrupted by the presence of suitable, reactive AA side-chain groups capable of donating an H-atom to neutralise a free radical. In this study, HPH was found to have the highest free histidine concentration of all FPHs in the current study (2.11 g/100 g protein), which could contribute to its high peroxidation inhibition values, where imidazole side chains present could bind ferrous ions [102]. HPH also had the highest total proline content (4.19 g/100 g protein). Proline is reported to reduce lipid peroxidation in systems as it is an active free radical scavenger [103]. Overall, the anti-lipid peroxidation IC_50_ values for all FPHs, including HPH, were higher than the IC_50_ values against lipid peroxidation reported previously for protein hydrolysates obtained from hake (*Merluccius merluccius*) heads (0.8–0.92 mg/mL) [27]. Variation in the production conditions along with an alternate method for evaluating peroxidation values may have contributed to the values obtained for HPH in this study and the study reported by Karoud et al. [27].

### 2.8. Anti-Diabetic Activity

Diabetes is a condition marked by an elevation in blood glucose levels caused by insulin dysfunction. Hyperglycaemia, or high blood sugar, can impair a number of bodily functions and, if uncontrolled, can result in potentially fatal conditions [104]. α-Amylase and α-glucosidase are important digestive enzymes responsible for the conversion of starch to oligosaccharides and glucose. They are therefore candidate therapeutic targets to treat diabetes [105], and there is considerable interest in finding natural substitutes that may reduce the action of starch-degrading enzymes while minimising adverse effects. This section explores the starch-degrading enzyme inhibitory effect of FPHs and the effect of SGID on inhibitory potential.

The FPH concentrations included in the enzyme inhibition studies were based on protein concentration. SPH demonstrated the most effective inhibition of α-amylase, with an IC_50_ value of 8.81 ± 0.01 mg/mL, followed by three other FPHs with comparable activity to each other: BWPH-B (IC_50_ = 14.94 ± 0.02 mg/mL), HPH (IC_50_ = 13.25 ± 0.01 mg/mL), and MPH (IC_50_ = 13.41 ± 0.03 mg/mL). At an IC_50_ value of 18.42 ± 0.01 mg/mL, BWPH-A exhibited the lowest degree of α-amylase inhibition. A common observation among all FPHs was the decline in activity after SGID, with some FPHs, such as SPH and HPH, losing their inhibitory potency entirely. Both FPHs from blue whiting saw a reduction in their IC_50_ values by ~50% (30.57 ± 0.00 and 31.44 ± 0.04 mg protein/mL for BWPH-A and BWPH-B, respectively, post-SGID), while MPH lost 80% potency following SGID (70.89 ± 0.01 mg protein/mL). In contrast, none of the FPHs inhibited α-glucosidase before or after SGID (up to a FPH concentration of 50 mg/mL).

From previous analysis of hydrolysates and the peptide sequences underpinning bioactivity, most peptides were in the size range of 2 to 20 AAs. A potential explanation for the reduction or loss of activity following SGID could be associated with the formation of more FAAs and a lower quantity of bioactive peptides capable of inhibiting the α-amylase enzyme. In a study conducted to assess the effects of yellowfin tuna (*Thunnus albacares)* hydrolysates against α-glucosidase, the highest inhibitory activity achieved (24.47%) was with FPH produced after the first hour of hydrolysis. As the FPH production time was increased, the α-glucosidase inhibitory effect decreased. These findings suggest that while partial hydrolysis could have generated peptides able to inhibit α-glucosidase, extensive hydrolysis might result in the breakdown of those peptides, leading to a decline in inhibitory activity [106]. Therefore, it is apparent that the kind of substrate, enzyme, and reaction conditions can impact this type of bioactivity.

Baba and colleagues identified roughly 22 peptide sequences consisting of 6–12 AAs from camel milk protein hydrolysates that could effectively inhibit or minimise the action of pancreatic α-amylase by binding to the enzyme’s catalytic/substrate-binding site [107]. In a different study conducted with peptides from Pinto beans (*Phaseolus vulgaris* cv. Pinto), the number of AAs in peptides displaying an α-amylase inhibitory effect also ranged from 6–12 [108]. Moreover, the size and sequence of the peptides, their AA composition also plays a significant role in determining bioactivity. Interestingly, Ngoh and Gan noted the presence of dipeptide repeats such as Proline-Proline, Cysteine-Cysteine, Leucine-Leucine, and Glycine-Glycine in peptide sequences against the activity of α-amylase [108]. Moreover, the most prevalent AA found in each peptide was proline. In the current study, proline is an abundant AA in the FPHs investigated, especially BWPH-B, HPH, and MPH, all of which had comparable IC_50_ values for α-amylase inhibition. Phenylalanine was the other AA that the authors identified at the N- and C-termini of inhibitory peptides. This AA is present in considerable quantities in SPH and BWPH-A. This association between specific AAs and α-amylase inhibition by peptides was corroborated by another study that identified albumin-derived α amylase inhibitory peptides and found that the most potent sequence was Lys-Leu-Pro-Gly-Phe, which contained proline within the sequence and phenylalanine at the end [109].

Little research has been conducted to date on the anti-diabetic properties of hydrolysates derived from fish by-products, and much less on their mechanism(s) of enzyme inhibition. The results from this study were almost the same as reported by Henriques and colleagues, who reported significant variation in the range of IC_50_ values (5.70 to 84.37 mg/mL) for α-amylase inhibition for hydrolysates from different fish sources [5]. Their IC_50_ results for whole fish blue whiting hydrolysates were around 18.51 ± 1.53 mg/mL, which was higher than BWPH-B (IC_50_ = 14.94 ± 0.02 mg/mL) but very similar to BWPH-A (IC_50_ = 18.42 ± 0.01 mg/mL) in the current study. Whole fish mackerel hydrolysate had an IC_50_ value of 84.37 ± 5.21 mg/mL in the study by Henriques and colleagues [5], which was even lower than the value for MPH post-SGID (IC_50_ = 70.89 ± 0.01 mg protein/mL) in the current study and significantly lower than for MPH pre-SGID, which had an IC_50_ of 13.41 ± 0.03 mg/mL, as mentioned earlier. A separate study carried out on underutilised fish such as Australian salmon, barracouta, and silver warehou reported no α-glucosidase inhibition and modest amounts of α-amylase inhibition [110]. The exact mechanism behind the interaction of peptides with the binding sites of enzymes such as α-amylase/glucosidase is still not fully understood. The FPHs that were produced in the present study did not display any inhibition against α-glucosidase. However, this does not rule out the possibility that use of a different combination of fish substrate and enzyme in FPH production may generate peptides with α-amylase and α-glucosidase inhibitory activity. Further research is needed on peptide-enzyme interactions to optimise the therapeutic potential of peptides from FPHs as natural dietary ingredients for the control of diabetes.

## 3. Materials and Methods

### 3.1. Materials and Reagents

Hydrolysates from blue whiting (BWPH-A and B) and sprat (SPH) were obtained in powdered form from BioMarine Ingredients Ireland Ltd. (Lough Egish Food Park, Castleblaney, Co., Monaghan, Ireland). Hake and mackerel processing wastes were obtained locally from ‘Eat More Fish’ (Ballybane Industrial Estate, Co., Galway, Ireland). Sodium acetate, hydrogen peroxide, phenol, neocuproine, 1,1-diphenyl-2-picrylhydrazyl (DPPH), linoleic acid, ammonium thiocyanate, ferric chloride, tricarboxylic acid (TCA), ammonium molybdate, pyrocatechol violet, ferrozine, salicylic acid, and 4-aminoantipyrene were purchased from Thermo Fisher Scientific, Loughborough, UK. All other reagents were obtained from Sigma–Aldrich (St. Louis, MO, USA), unless stated otherwise. p-nitrophenyl-α-D-glucopyranoside (p-NPG) was purchased from Carbosynth Ltd., Compton, CA, USA. Ultrapure water obtained from the Milli-Q Plus system (Millipore Corporation, Milford, MA, USA) was used for all solution preparations. Pierce™ BCA Rapid Gold Protein Kit was obtained from Fisher Scientific, Dublin, Ireland, part of Thermo Fisher Scientific.

### 3.2. Preparation of FPH

The complete blue whiting (*Micromesistius poutassou*), frozen immediately after being caught in the Northeast Atlantic, was used to generate the studied FPHs (Biomarine Ingredients Ireland Ltd., Monaghan, Ireland). The fish was thawed, and the bones were separated using a vibrational sieve. The residual raw material was then hydrolysed with enzymes of microbial origin. The insoluble partly hydrolysed protein fraction (BWPH-A) and water-soluble protein hydrolysate fraction (BWPH-B) were separated and spray-dried to prevent thermal damage to the protein. The reaction conditions for the generation of FPHs are similar to those previously reported [13]. The partially hydrolysed sprat (*Sprattus sprattus*) insoluble fraction (SPH) was produced under similar proprietary conditions.

### 3.3. Generation of In-House FPH

Hake and mackerel processing wastes were obtained locally from ‘Eat More Fish’ (Ballybane Industrial Estate, Co., Galway, Ireland). Two microbial-based enzymes—Alcalase^®^ and Flavourzyme— were used to perform hydrolysis on the fish processing wastes under similar conditions, and the release of soluble protein was thereafter quantified using a protein kit (Pierce™ BCA Rapid Gold Protein kit). The fish minces were prepared freshly before use and were adjusted to a final consistency of 6% (*w*/*v*). Hydrolysis was initiated by adding the enzymes to the mince at pH 7.04 and allowing the mixture to incubate at 50 °C for 4 h. Replicate controls were incubated alongside, to which buffer was added instead of the enzyme. Post hydrolysis, the samples were heat-treated at 90 °C for 20 min to inactivate the enzymes and allowed to cool on ice thereafter. The soluble protein content of test samples was quantified by the protein kit using bovine serum albumin (BSA) as the standard. FPHs with the highest soluble protein content were chosen and freeze dried for further analysis.

### 3.4. Proximate Composition of FPH

The total protein content was evaluated using a LECO FP-628 Analyser (Teagasc Food Research Centre, Ashtown, Ireland) based on the Dumas method and in accordance with AOAC method 992.15, 1990. Nitrogen was converted to crude protein percent using a ratio of 6.25.

Moisture content was determined gravimetrically after drying the powders (in triplicate) at 65 °C for 24 h and then measuring the dry weight of the sample.

Ash content was then analysed by subjecting the dry samples to heating from 105 °C to 250 °C for 30 min at a heating rate of 10 °C/min and then to 550 °C for 2 h at a heating rate of 20 °C/min (Memmert GmbH, Schwabach, Germany).

Fat was determined using the Oracle NMR rapid fat analyser (CEM Corporation, USA) according to AOAC method 2008.06. Pre-drying was performed using Smart 6 (CEM Corporation, Charlotte, NC, USA) according to AOAC Official Methods 985.14, 1990.

Carbohydrate content was determined using the modified Dubois method [111]. Equal volumes (50 µL) of sample and 5% (*w*/*w*) phenol were mixed and allowed to cool at 4 °C for 10–15 min. To the mixture, 250 µL of conc H_2_SO_4_ was added, and the tubes were boiled for 5 min. After cooling, the absorbance of samples was read at 490 nm using a spectrophotometer (BioTek PowerWave XS2, Winooski, VE, USA).

### 3.5. Functional Properties

#### 3.5.1. Solubility and Turbidity

A pH range of 2.0–10.0 was used to investigate the solubility of FPHs. Briefly, 25 mL of milliQ water was used to dissolve 0.25 g of each sample, and 1M HCl or NaOH solutions was used to adjust the pH. The mixture was agitated for 15 min, followed by 10 min of centrifugation at 7000× *g* at 4 °C. The protein content of the supernatant was assessed using the BCA protein kit, where BSA was used as the standard. The solubility was determined by the following equation:

(1) $$\mathrm{Solubility}\;(\%)=\frac{\mathrm{Protein}\mathrm{content}\mathrm{in}\mathrm{supenatant}}{\mathrm{Total}\mathrm{protein}\mathrm{content}\mathrm{in}\mathrm{FPH}}\times 100$$

where the total protein content in FPH was obtained using LECO analysis.

Turbidity was evaluated by preparing FPHs (1% *w*/*v*) in milliQ water and adjusting their pH from 2–10 with 1M HCl and NaOH. The OD of the samples was measured in a spectrophotometer at a wavelength of 660 nm. MilliQ water was used as the blank.

#### 3.5.2. Water Holding Capacity (WHC) and Oil Holding Capacity (OHC)

To evaluate the WHC and OHC of FPHs, 50 mL of milliQ water and vegetable oil were thoroughly mixed with 1 g of sample each (in duplicates). The samples were then centrifuged at 4000× *g* for 30 min after standing at RT for 30 min. The volume of free water or oil observed was later converted to grams using the density of the substance. Absorption was calculated as the amount of water or oil that was absorbed per gramme of sample.

#### 3.5.3. Determination of Bulk Density

Approximately 2 g of each FPH was weighed and placed into a 25 mL graduated cylinder. The cylinder was gently tapped 20 times, and the volume was recorded. The bulk density was expressed as g mL^−1^ of FPH.

#### 3.5.4. Foaming Capacity (FC) and Stability (FS)

Following the method employed by [112], 0.25 g of each FPH was dissolved in 25 mL of milliQ water, and the pH adjusted to 2, 4, 6, 8, and 10. After 3 min of whipping, the solution was poured into a 100 mL graduated cylinder. The total sample volume was taken at 0 min for FC and up to 5 min for FS. Both FC and FS were calculated as follows:

(2) $$\mathrm{FC}\;(\%)=\frac{\left(A-B\right)}{B}\times 100$$

(3) $$\mathrm{FS}\;(\%)=\frac{\left(C-B\right)}{B}\times 100$$

where A is the volume after whipping (mL), B is the volume before whipping (mL), and C is the volume after standing (mL).

#### 3.5.5. Emulsifying Activity and Stability

Different concentrations (0.5, 1, and 2%) of FPHs were dissolved in 3 mL of milliQ water, and to each solution, 1 mL of vegetable oil was added. The mixture was homogenised at high speed for 1 min using a vortex. A volume of 50 µL was taken from the mixture immediately after and mixed with 0.1% sodium dodecyl sulphate (SDS) solution. The absorbance was read with a spectrophotometer at a wavelength of 500 nm. After 10 min, 50 µL of the same solution was taken and mixed with 0.1% SDS, and the absorbance was recorded. The emulsion activity index (EAI) and emulsion stability index (ESI) were calculated according to the following equation:

(4) $$\mathrm{EAI}\;(m^{2}/g)=\frac{2\times 2.303\times A500}{\emptyset \times sample\;weight(g)}\times 100$$

(5) $$\mathrm{ESI}\;(\%)=100-\frac{EAI\;A0-EAI\;A10}{EAI\;A0}\times 100$$

where A500 = absorbance at 500 nm, ø = oil volumetric fraction (0.25), A0 = absorbance at 0 min, and A10 = absorbance at 10 min.

### 3.6. Amino Acid Analysis

Analysis of FAA and TAA was conducted on dried powders by Teagasc (Moorepark, Ireland) according to the method described by [113]. Equal parts of the sample and tri-chloroacetic acid (24% *w*/*v*) were combined to deproteinise the samples. After standing for 10 min, the contents were centrifuged for an additional 10 min at 15,000 rpm. Supernatants were eliminated, and samples were diluted 1 in 2 with norleucine, the internal standard, to produce a final concentration of 125 nmol/mL norleucine. A Jeol JLC-500/V amino acid analyser (Jeol (UK) Ltd., Garden City, Herts, UK) equipped with a Jeol Na+ high perfomance cation exchange column was used to measure AAs.

Based on the AA composition of the FPHs, a chemical score was determined. It is the ratio of EAA in the test protein to a standard protein as defined by FAO/WHO/UNU for adult humans [48] and NRC for carp [49]. The protein efficiency ratio (PER) of the FPHs was predicted according to the equations described by [50]:

1 = −0.684 + 0.456[Leu] − 0.047 [Pro](6)

2 = −0.468 + 0.456 [Leu] − 0.104 [Tyr](7)

3 = −1.816 + 0.435 [Met] + 0.780 [Leu] + 0.211 [His] − 0.944 [Tyr](8)

4 = 0.08084 [A] − 0.1094(9)

5 = 0.06320 [B] − 0.1539(10)

where A = Thr + Val + Met + Ile + Leu + Phe + Lys and B = A + His + Arg + Tyr.

The biological value (BV) was estimated as 49.09 + 10.53 [PER (Equation (9)].

### 3.7. Scanning Electron Microscopy

FPHs were observed under a scanning electron microscope (Hitachi S2600N, Krefeld, Germany). Prior to examination, the specimens were gold coated (Quorum Q150R ES+, Sussex, UK) and then examined under a magnification of 450×, at an accelerating voltage of 15.0 kV.

### 3.8. Fourier Transform Infrared (FTIR) Analysis

A small quantity of dried FPH was combined with 100 mg potassium bromide (KBr) before being placed on the iD7 ATR accessory paired with the Thermo Scientific™ Nicolet™ iSTM5 FT-IR Spectrometer. FTIR spectra between 400 and 4000 cm^−1^ with a resolution of 2 cm^−1^ were examined in the absorbance mode. The spectra were analysed using OMNIC software (Thermo Fisher, Waltham, MA, USA, v. 9.9.509).

### 3.9. Simulated Gastrointestinal Digestion (SGID)

An in vitro digestion model system was employed, utilising enzymes that are commonly found in the upper GI tract of humans [114]. Pepsin (40 g/kg, protein basis) was added after the pH of the FPHs (50 g/L in milliQ water) had been adjusted to 2.0 with 1M HCl. Later, the pH of the solution was changed to 5.3 with 0.9 M NaHCO_3_ after 1 h of incubation at 37 °C. After pH was raised to 7.5 (1 M NaOH) and pancreatin (40 g/kg, protein basis) added, the mixture was incubated at 37 °C for 2 h. Both enzymatic reactions were terminated by heating in boiling water for 10 min. The digests were allowed to cool to RT before being centrifuged at 11,000× *g* for 15 min. The supernatant was obtained, and the protein concentration was evaluated by the Pierce™ BCA Rapid Gold Protein Kit. The test samples were then frozen at −20 °C for further analysis.

### 3.10. Estimation of Degree of Hydrolysis

DH was measured using a formol titration method [115]. FPHs (~1.5 g) were dissolved in milliQ water, and the volume was made up to 50 mL. The pH of the solution was then adjusted to 7.0 with 0.1 M NaOH, followed by the addition of 10 mL of formaldehyde (36.5% *v*/*v*). The solution was allowed to stir at RT for 5 min, and then the final titration was conducted with the end point as 8.5 with 0.1 M NaOH. The % of FAA groups was calculated using the following equation:

(11) $$\mathrm{AA}\;\mathrm{groups}\;(\%)=\frac{V\;X\;C\;X\;14.007}{S}\times 100$$

where V (mL) is the volume of NaOH, C is the concentration of NaOH, and W (g) is the weight of the sample.

The DH was calculated by the following equation:

(12) $$\mathrm{DH}\;(\%)=\frac{\%free\;amino\;groups}{\%total\;nitrogen}$$

where the total nitrogen of the sample was obtained with LECO analysis.

### 3.11. Determination of Antioxidant Activity

#### 3.11.1. Ferric Reducing Antioxidant Power (FRAP)

A revised adaptation was used to assess the FRAP of FPHs [116]. Sodium acetate buffer (pH 3.6), 40 mM 2,4,6-Tris(2-pyridyl)-s-triazine (TPTZ) solution, and 20 mM aqueous FeCl_3_ were combined in a 1:1:10 ratio and pre-incubated at 37 °C to create the FRAP reagent. Tests and standards (in triplicates) were combined with 0.18 mL of the reagent, and the mixture was then incubated at 37 °C for 40 min. The absorbance was measured at 590 nm, and the findings were represented in terms of µmoles of Trolox equivalents per 100 milligrammes of protein (µmoles of TE/100 mg of protein).

#### 3.11.2. Potassium Ferricyanide Reducing Antioxidant Power (P-FRAP)

A microplate-based adaptation was used to assess the antioxidant capacity of FPHs to reduce potassium ferricyanide [117]. Equal parts of 0.01 mL of tests and standards (in triplicates) were mixed with 0.03 mL of 0.2 M sodium phosphate buffer (pH 6.6) and 1% potassium ferricyanide. After 20 min of incubation at 50 °C, 0.03 mL of 10% trichloroacetic acid was added to the microplate. After diluting the mixture with 0.1 mL of milliQ water, 0.02 mL of 0.1% FeCl_3_ were added to each test and standard. The findings were represented as mg AAE/100 g DW sample after the absorbance at 700 nm was measured.

#### 3.11.3. Cupric Ion Reducing Antioxidant Capacity (CUPRAC)

The CUPRAC test was carried out by employing the method suggested by [118]. The tests and standards (77 µL in triplicates) contained equal volumes (70 µL) of 10 mM CuCl_2_, 1M ammonium acetate buffer (pH 7.0), and 7.5 mM neocuproine (in 96% ethanol). The mixture was incubated for 30 min at 50 °C, and the absorbance at 450 nm was measured. Reducing power was expressed in terms of milligramme ascorbic acid equivalents per 100 g of sample dry weight (mg AAE/100 g DW sample).

#### 3.11.4. Phosphomolybdenum Assay

According to the procedure outlined by [119], in 0.6 M sulphuric acid, 28 mM sodium phosphate and 4 mM ammonium molybdate were combined to create the working reagent. 0.1 mL of tests and standards (in triplicates) were mixed with 1 mL of the reagent, and the mixture was then incubated at 95 °C for 30 min. After the preparations had cooled, their absorbance at 695 nm was measured, and the activity was represented in milligrammes of ascorbic acid equivalents for every 100 g of dry weight of the sample (mg AAE/100 g DW sample).

#### 3.11.5. Cupric Ion Chelating Activity

The cupric ion (Cu^2+^)-chelating activity was determined using a slightly modified approach from [120]. 30 µL of 2 mM CuSO_4_.5H_2_O solution, 8.5 µL of 2 mM pyrocatechol violet, and 200 µL of sodium acetate buffer (50 mM, pH 6.0) were added to each well of a 96-well microplate. 30 µL of tests and standards at various concentrations were added, and the mixture was shaken for 10 min and then incubated for 10 more at 25 °C. Afterwards, the absorbance at 632 nm was measured, and the percentage of metal chelation activity was estimated using the formula:

(13) $$Chelating\;activity\left(\%\right)=\frac{Acontrol-Asample}{Acontrol}\times 100$$

where Acontrol is the absorbance of the sample-free control and Asample is the absorbance of the sample in the presence of the chelator.

#### 3.11.6. Iron Chelating Activity

The Fe^2+^ chelating activity of the samples was determined following the method published by [121]. Samples (30 µL) were mixed with 250 µL of 100 mM sodium acetate buffer (pH 4.9). To this mixture, 30 µL FeCl_2_ (0.2 mg/mL) was added and allowed to incubate at RT in the dark for 30 min. Finally, 12.5 µL of a 40 mM Ferrozine solution was added. The absorbance was recorded at 562 nm. The percentage of metal chelation activity was estimated using the same formula as in Section 3.11.5.

#### 3.11.7. DPPH Radical Scavenging Assay

The DPPH radical scavenging activity of the samples was investigated following the method outlined by [122]. The samples (100 µL) were combined with 100 µL of 0.2 mM DPPH solution prepared in methanol. After the mixture was left to incubate for 30 min at RT, the absorbance was recorded at 517 nm using a spectrophotometer. The following equation was used to calculate the radical scavenging activity:

(14) $$Scavenging\;activity\left(\%\right)=\frac{Acontrol-Asample}{Acontrol}\times 100$$

where Acontrol is the absorbance of the sample-free control and Asample is the absorbance of the sample in the presence of the scavenger.

#### 3.11.8. ABTS Radical Scavenging Assay

The ABTS radical scavenging activity of the FPHs was evaluated following the method of [123]. The working ABTS reagent was made by mixing an equal amount of 2.45 mM ammonium persulphate solution with a 7 mM solution of ABTS reagent generated in acetic acid buffer (0.1 M, pH 4.5). The mixture was then left to incubate in the dark for 12–16 h at RT. The following day, 2 mL of ABTS reagent was diluted in buffer to attain an absorbance of 0.74 ± 0.03 at a wavelength of 734 nm. The samples (50 µL) were combined with the assay reagent (150 µL), and the mixture was left to incubate in the dark for 7 min. Later, the absorbance was measured at 734 nm, and the scavenging activity was estimated using the same formula as in Section 3.11.7.

#### 3.11.9. Hydroxyl Radical Scavenging Assay

A microplate adaptation for evaluating the hydroxyl radical scavenging activity of FPHs was conducted [124]. Equal amounts (10 µL) of 6 mM FeSO_4_ and 6 mM H_2_O_2_ solution were mixed with samples at different dilutions (40 µL), and the resulting mixture was then brought up to a volume of 190 µL using milliQ water. After a few min of gently shaking the plate, 10 µL of 6 mM salicylic acid was added to each well, and the mixture was then incubated for 30 min at 37 °C. Following the measurement of absorbance at 510 nm, the scavenging activity was calculated using the same formula as in Section 3.11.7.

#### 3.11.10. Hydrogen Peroxide Radical Scavenging Assay

The hydrogen peroxide radical scavenging activity of FPHs was assessed using a microplate-based adaptation of [125]. The test sample (70 µL), phenol solution (12 mM, 70 µL), 4-aminoantipyrene (0.5 mM, 20 µL), H_2_O_2_ (0.7 mM, 32 µL), and HRP (1 U/mL, 8 µL) generated in phosphate buffer (84 mM, pH 7.0) made up the reaction mixture in a single well. After mixing the components, the mixture was left to incubate for 30 min at 37 °C. A spectrophotometer was then used to measure the absorbance at 504 nm. The scavenging activity was estimated using the same formula as in Section 3.11.7.

#### 3.11.11. Superoxide Radical Scavenging Assay

The method of [126], modified by [127], was used to assess the superoxide radical scavenging activity of FPHs. After combining the samples (50 µL) with 140 µL of 50 mM Tris-HCl buffer (1 mM EDTA, pH 7.4), each well of the microplate was given 20 µL of a 60 mM pyrogallol solution (in 1 mM HCl). After 5 min of incubation at 37 °C, the absorbance of the mixture was measured at 325 nm with a spectrophotometer. The scavenging activity was estimated using the same formula as in Section 3.11.7.

#### 3.11.12. Lipid Peroxidation Inhibition Assay

The inhibition of lipid peroxidation was estimated by the ferric thiocyanate method, modified by [128]. Equal volumes (50 µL) of sample, linoleic acid (50% *v*/*v*) in ethanol, ammonium thiocyanate (10% NH_4_SCN), and FeCl_2_ (2 mM) were mixed and allowed to incubate at 37 °C for 1 h. The absorbance of the mixture was measured at 500 nm with a spectrophotometer. The following formula was used to determine the inhibition:

(15) $$Lipid\;peroxidation\;inhibition\left(\%\right)=\frac{Acontrol-Asample}{Acontrol}\times 100$$

where Acontrol is the absorbance of the sample-free control and Asample is the absorbance of the sample in the presence of the inhibitor.

### 3.12. Determination of Antidiabetic Activity

#### 3.12.1. α-Amylase Inhibition Assay

The FPHs were tested for their activity against α-amylase based on a microtiter plate method described by [129]. Each hydrolysate (100 µL) was pre-incubated with the enzyme (1 U/mL, 100 µL) for 10 min at 37 °C, after which a starch solution (1% *w*/*v*, 100 µL) was added to the mixture and allowed to stand at 37 °C for 10 min. The reaction was stopped by adding 100 µL of 3,5-dinitrosalicylic acid, and the tubes were boiled in a water bath for 5 min. After cooling, the mixture was diluted by adding 1 mL of milliQ water, and the contents were transferred to a 96-well microtiter plate, after which the absorbance was read at 540 nm. Inhibition of α-amylase was calculated using the following equation:

(16) $$\alpha -amylase\;inhibition\left(\%\right)=\frac{Acontrol-Asample}{Acontrol}\times 100$$

where Acontrol is the absorbance of the sample-free control and Asample is the absorbance of the sample in the presence of the inhibitor.

#### 3.12.2. α-Glucosidase Inhibition Assay

Inhibition against α-glucosidase was based on a slightly modified microtiter plate method described by [130]. The samples (50 µL) were mixed with the α-glucosidase enzyme (1 U/mL), and the volume was brought up to 100 µL with phosphate buffer (50 mM, pH 6.5). After incubating the mixture for 5 min at RT, the substrate p-nitrophenyl-α-D-glucopyranoside (p-NPG) was added (50 µL, 3 mM). This mixture was further allowed to incubate at RT for 15 min, after which 1 M sodium carbonate (100 µL) was used to stop the reaction. The absorbance was measured at 405 nm.

### 3.13. Statistical Analysis

The experimental data was presented as mean values ± standard deviation of duplicates or triplicates. Statistical analysis of the data was carried out using the mean, standard deviation, t-test (Student’s and Welch’s), and one-way ANOVA, with significance at *p* < 0.05. Analysis and graphical representation were carried out using Microsoft Excel 2023 (Microsoft, Washington, DC, USA).

## 4. Conclusions

Hydrolysis of four different fish species—blue whiting, sprat, hake, and mackerel yielded five different FPHs; BWPH-A and BWPH-B were the partly and extensively hydrolysed fractions from blue whiting. SPH was partially hydrolysed from sprat, and HPH and MPH were more extensively hydrolysed from hake and mackerel, respectively. The FPHs were high in protein content and abundant in EAAs, which suggests their potential nutritional and health benefits. The antioxidant and anti-diabetic activities of the FPHs were strongly affected by the peptide size and AA composition. The hydrolysates from sprat, hake, and mackerel exhibited the greatest bioactivity, which could be a result of the respective by-product characteristics as well as the proteins present and their response to the enzymes used in the production methods. These findings suggest that the FPHs are promising sources of bioactive peptides and can be utilised as dietary supplements with nutraceutical potential. Their inclusion in food formulations may prolong the shelf-life of products and keep them fresh by delaying oxidation. Investigation of the FPHs, or peptides therefrom, in pharmaceutical markets, based on the results of their in vitro studies, is also a future possibility. However, such applications would require extensive in vivo experiments to evaluate bioavailability, safety, and efficacy.

## Figures and Tables

**Table 1:** Proximate composition of FPHs: BWPH-A: blue whiting protein hydrolysate A; BWPH-B: blue whiting protein hydrolysate B; SPH: sprat protein hydrolysate; HPH: hake protein hydrolysate; MPH: mackerel protein hydrolysate. Results are expressed as mean values ± SD (*n* = 3). * Different letters in the same column indicate significantly different means (*p* < 0.05).

Source	Dry Matter (%)	Protein (%)	Moisture (%)	Ash (%)	Lipid (%)	Sugar (%)
BWPH-A	96.2 ± 0.12 ^a,^*	73.24 ± 0.02 ^d^	3.8 ± 0.12 ^d^	7.59 ± 1.18 ^c^	8.04 ± 0.8 ^b^	0.65 ± 0.00 ^a^
BWPH-B	96.25 ± 0.02 ^a^	89.31 ± 0.11 ^a^	3.75 ± 0.02 ^d^	10.06 ± 0.62 ^b^	0.18 ± 0.00 ^c^	0.38 ± 0.01 ^b^
SPH	95.4 ± 0.18 ^b^	59.34 ± 0.19 ^e^	4.56 ± 0.18 ^c^	9.14 ± 0.08 ^b,c^	14.63 ± 0.77 ^a^	0.16 ± 0.01 ^c^
HPH	94.62 ± 0.15 ^c^	77.56 ± 0.04 ^b^	5.38 ± 0.15 ^b^	11.59 ± 0.21 ^b^	0.8 ± 0.09 ^c^	0.88 ± 0.09 ^a^
MPH	91.25 ± 0.21 ^d^	74.76 ± 0.1 ^c^	8.75 ± 0.21 ^a^	12.85 ± 0.14 ^a^	0.71 ± 0.16 ^c^	0.64 ± 0.12 ^a^

**Table 2:** Water and oil holding capacities and bulk density of FPHs: BWPH-A: blue whiting protein hydrolysate A; BWPH-B: blue whiting protein hydrolysate B; SPH: sprat protein hydrolysate, HPH: hake protein hydrolysate; MPH: mackerel protein hydrolysate; WHC: water holding capacity; OHC: oil holding capacity; FPH: fish protein hydrolysate. Results are expressed as mean values ± SD (*n* = 3).

Source	WHC (g Water/g FPH)	OHC (g Oil/g FPH)	Bulk Density (g mL^−1^)
BWPH-A	3.5 ± 0.71 ^a^	3.62 ± 0.08 ^a^	0.63 ± 0.03 ^a,c,e^
BWPH-B	1.25 ± 1.06 ^b^	2.53 ± 1.63 ^b^	0.76 ± 0.01 ^b^
SPH	2.25 ± 1.06 ^b^	2.53 ± 0.33 ^c^	0.70 ± 0.01 ^c^
HPH	0.15 ± 0.07 ^c^	3.17 ± 0.07 ^d^	0.72 ± 0.01 ^c,d^
MPH	0.75 ± 0.35 ^d^	3.58 ± 0.14 ^a,d^	0.60 ± 0.04 ^e^

Note: Different letters in the same column indicate significantly different means (*p* <0.05).

**Table 3:** Emulsifying properties of FPHs at different concentrations: BWPH-A: blue whiting protein hydrolysate A; BWPH-B: blue whiting protein hydrolysate B; SPH: sprat protein hydrolysate; HPH: hake protein hydrolysate; MPH: mackerel protein hydrolysate. Results are expressed as mean values ± SD (*n* = 3).

Source	BWPH-A	BWPH-B	SPH	HPH	MPH
Emulsifying activity index (m^2^/g)					
0.5	68.48 ± 0.53 ^a^*	108.09 ± 3.24 ^a^	61.11 ± 2.13 ^a^	104.71 ± 19.18 ^a^	80.45 ± 0.53 ^a^
1	49.95 ± 2.56 ^b^	74.11 ± 6.27 ^b^	35.42 ± 0.71 ^b^	82.29 ± 11.11 ^a^	61.00 ± 0.35 ^b^
2	27.84 ± 0.64 ^c^	39.71 ± 0.71 ^c^	21.49 ± 0.31 ^c^	31.01 ± 1.71 ^b^	23.75 ± 0.94 ^c^
Emulsifying stability index (%)					
0.5	61.89 ± 2.61 ^a^	37.82 ± 3.14 ^a^	70.88 ± 1.37 ^a^	76.99 ± 13.52 ^a^	51.92 ± 2.83 ^a^
1	64.49 ± 4.51 ^a^	50.94 ± 1.98 ^b^	87.31 ± 2.39 ^b^	96.44 ± 10.28 ^a^	62.75 ± 1.65 ^b^
2	64.74 ± 2.09 ^a^	58.77 ± 1.23 ^c^	78.09 ± 0.88 ^c^	98.12 ± 3.38 ^a^	63.41 ± 1.91 ^b^

* Different letters in the same column indicate significantly different means (*p* < 0.05).

**Table 4:** Amino acid composition of FPHs (g/100 g protein): BWPH-A: blue whiting protein hydrolysate A; BWPH-B: blue whiting protein hydrolysate B; SPH: sprat protein hydrolysate; HPH: hake protein hydrolysate; MPH: mackerel protein hydrolysate; AA: amino acid; TAA: total amino acids; FAA: free amino acids; EAA: essential amino acids; NEAA: non-essential amino acids.

	BWPH-A		BWPH-B		SPH		HPH		MPH	
EAA	TAA	FAA	TAA	FAA	TAA	FAA	TAA	FAA	TAA	FAA
Threonine	3.17	0.07	3.14	0.26	2.52	0.04	3.09	1.26	3.05	1.33
Valine	4.11	0.10	3.40	0.56	3.46	0.12	3.39	1.49	3.46	1.78
Met + Cys ^a^	0.19	0.03	0.15	0.44	0.19	-	0.13	1.23	0.15	1.25
Isoleucine	3.84	0.06	2.80	0.25	2.98	0.06	2.84	1.02	3.00	1.28
Leucine	6.24	0.24	5.87	1.00	4.66	0.21	5.14	2.75	5.17	2.86
Phe + Tyr ^b^	6.58	0.24	4.32	1.22	4.93	0.16	4.78	2.24	4.97	2.36
Histidine	1.93	0.26	2.28	1.63	1.46	0.19	1.50	2.11	1.45	1.95
Lysine	5.55	0.06	7.41	0.37	3.98	0.09	5.05	1.07	5.14	1.36
Tryptophan	-	-	-	-	-	-	-	2.33	-	2.77
Arginine	4.88	0.10	5.48	0.50	3.58	0.20	5.20	1.62	5.02	11.72
Serine	3.14	0.05	3.62	0.21	2.29	0.05	3.31	1.07	3.31	11.21
Glutamic Acid	10.72	0.10	15.31	0.57	8.25	0.07	11.90	1.75	11.94	2.21
Aspartic Acid	9.50	0.03	8.69	0.16	5.89	0.06	7.07	0.81	7.20	1.20
Glycine	3.59	0.03	6.02	0.12	3.20	0.02	7.42	0.66	6.63	0.83
Alanine	4.24	0.13	5.67	0.45	3.28	0.15	5.28	1.82	5.05	2.18
Proline	2.87	-	3.52	-	2.24	-	4.19	-	3.89	0.20
∑AA	70.55	1.59	77.69	8.15	52.91	1.53	70.30	24.63	69.44	27.95
∑EAA	31.60		29.37		24.18		25.92		26.39	
∑NEAA	38.95		48.32		28.73		44.38		43.04	
∑EAA/∑AA%	44.79		37.80		45.70		36.88		38.01	
∑EAA/∑NEAA	0.81		0.61		0.84		0.58		0.61	

^a^ Methionine and Cysteine are grouped together for scoring purposes since the latter can be synthesised from the former. ^b^ Phenylalanine and Tyrosine are grouped together for similar purposes.

**Table 5:** Chemical score of FPHs (g/100 g protein) compared with FAO/WHO and NRC reference proteins: BWPH-A: blue whiting protein hydrolysate A; BWPH-B: blue whiting protein hydrolysate B; SPH: sprat protein hydrolysate; HPH: hake protein hydrolysate; MPH: Mackerel protein hydrolysate; AA: amino acid; C1: chemical score calculated with reference protein 1; C2: chemical score calculated with reference protein 2.

			BWPH-A		BWPH-B		SPH		HPH		MPH	
Essential AA	Ref Protein 1 ^a^	Ref Protein 2 ^b^	C1	C2	C1	C2	C1	C2	C1	C2	C1	C2
Histidine	1.6	2.1	1.21	0.92	1.42	1.08	0.91	0.69	0.94	0.71	0.91	0.69
Isoleucine	1.3	2.5	2.96	1.54	2.15	1.12	2.29	1.19	2.18	1.14	2.31	1.20
Leucine	1.9	3.3	3.28	1.89	3.09	1.78	2.45	1.41	2.71	1.56	2.72	1.57
Phe + Tyr	-	6.5	-	1.01	-	0.66	-	0.76	-	0.74	-	0.76
Threonine	0.9	3.9	3.52	0.81	3.49	0.81	2.80	0.65	3.43	0.79	3.39	0.78
Valine	1.3	3.6	3.16	1.14	2.61	0.94	2.66	0.96	2.61	0.94	2.66	0.96
Met + Cys	1.7	3.1	0.11	0.06	0.09	0.05	0.11	0.06	0.08	0.04	0.09	0.05
Lysine	1.6	5.7	3.47	0.97	4.63	1.30	2.49	0.70	3.16	0.89	3.21	0.90

^a^ recommended EAA requirement for adults (FAO/WHO, 1990 [48]); ^b^ recommended EAA requirements for carp (NRC, 1993 [49]).

**Table 6:** Protein efficiency ratio (PER) and biological value (BV) of FPHs: BWPH-A: blue whiting protein hydrolysate A; BWPH-B: blue whiting protein hydrolysate B; SPH: sprat protein hydrolysate; HPH: hake protein hydrolysate; MPH: mackerel protein hydrolysate.

Equation	Equation	BWPH-A	BWPH-B	SPH	HPH	MPH
1	−0.684 + 0.456 [Leu] − 0.047 [Pro]	2.03	1.83	1.34	1.46	1.49
2	−0.468 + 0.456 [Leu] − 0.104 [Tyr]	2.07	2.04	1.44	1.67	1.67
3	−1.816 + 0.435 [Met] + 0.780 [Leu] + 0.211 [His] − 0.944 [Tyr]	0.71	1.79	0.24	0.69	0.55
4	0.08084 [A] − 0.1094	2.16	2.06	1.67	1.81	1.84
5	0.06320 [B] − 0.1539	2.15	2.05	1.56	1.81	1.79
BV	49.09 + 10.53 (PER)	70.84	70.56	64.27	66.68	66.63

**Table 7:** Antioxidant activities of FPHs before and after SGID: BWPH-A: blue whiting protein hydrolysate A; BWPH-A_SGID: blue whiting protein hydrolysate-A post SGID; BWPH-B: blue whiting protein hydrolysate B; BWPH-B_SGID: blue whiting protein hydrolysate-B post SGID; SPH: sprat protein hydrolysate; SPH_SGID: sprat protein hydrolysate post SGID; HPH: hake protein hydrolysate; HPH_SGID: hake protein hydrolysate post SGID; MPH: mackerel protein hydrolysate; MPH_SGID: mackerel protein hydrolysate post SGID. Data represent mean values ± SD (*n* = 3). Different letters in a column indicate significantly different means (*p* < 0.05).

Antioxidant Reducing Power (µmoles Equivalent/g Protein)				
Hydrolysate	FRAP	P-FRAP	CupRAC	PMD
BWPH_A	23.20 ± 8.29 ^a,b,c,e^	19.99 ± 1.92 ^e,f^	69.77 ± 1.93 ^g^	223.86 ± 5.82 ^g,h,i,j,k^
BWPH-A_SGID	33.86 ± 1.24 ^b,e^	16.47 ± 1.05 ^d^	56.39 ± 6.40 ^f,g^	207.75 ± 15.01 ^a,g^
BWPH_B	31.84 ± 2.38 ^b,e^	13.22 ± 0.63 ^d^	40.59 ± 0.32 ^f^	84.28 ± 10.48 ^f^
BWPH-B_SGID	33.01 ± 0.65 ^e^	15.69 ± 0.26 ^b,e^	27.62 ± 1.32 ^e^	145.61 ± 9.08 ^c,d,e^
SPH	107.15 ± 4.17 ^d^	91.67 ± 17.93 ^c^	293.18 ± 53.54 ^d^	501.47 ± 3.90 ^b^
SPH_SGID	100.49 ± 22.15 ^d^	38.61 ± 6.82 ^a^	173.47 ± 43.64 ^a,b^	269.74 ± 48.44 ^a,h^
HPH	24.85 ± 0.91 ^c^	18.16 ± 1.22 ^b,f^	36.07 ± 8.74 ^c,e,f^	192.60 ± 1.82 ^a^
HPH_SGID	34.79 ± 0.89 ^b^	7.79 ± 4.81 ^b,d^	52.09 ± 3.53 ^c^	203.55 ± 15.98 ^a,c,i^
MPH	35.85 ± 0.64 ^b^	34.30 ± 0.16 ^a^	75.67 ± 1.91 ^b^	216.81 ± 16.48 ^a,c,j^
MPH_SGID	43.99 ± 2.32 ^a^	31.75 ± 2.51 ^a^	64.92 ± 5.29 ^a,g^	204.93 ± 14.25 ^a,c,k^

**Table 8:** Metal ion chelating activities of FPHs before and after SGID: BWPH-A: blue whiting protein hydrolysate A; BWPH-A_SGID: blue whiting protein hydrolysate-A post SGID; BWPH-B: blue whiting protein hydrolysate B; BWPH-B_SGID: blue whiting protein hydrolysate-B post SGID; SPH: sprat protein hydrolysate; SPH_SGID: sprat protein hydrolysate post SGID; HPH: hake protein hydrolysate; HPH_SGID: hake protein hydrolysate post SGID; MPH: mackerel protein hydrolysate; MPH_SGID: mackerel protein hydrolysate post SGID. Data represent mean values ± SD (*n* = 3). Different letters in a column indicate significantly different mean values (*p* < 0.05). The results are expressed as IC_50_ (concentration of FPHs that chelate metal ions by 50%).

	Metal ion Chelating Activity (IC_50_ mg/mL Protein)	
Hydrolysate	Cu^2+^	Fe^2+^
BWPH_A	1.93 ± 0.06 ^e^	1.89 ± 0.24 ^c^
BWPH-A_SGID	1.43 ± 0.09 ^d^	21.30 ± 4.33 ^a,b,d,e^
BWPH_B	3.49 ± 0.23 ^c^	46.34 ± 0.84 ^f^
BWPH-B_SGID	0.86 ± 0.04 ^a^	27.18 ± 0.93 ^e^
SPH	1.60 ± 0.09 ^b,d^	5.18 ± 1.85 ^c^
SPH_SGID	1.75 ± 0.09 ^b,e^	12.52 ± 1.17 ^b,d^
HPH	0.66 ± 0.26 ^a^	4.67 ± 0.33 ^c^
HPH_SGID	0.78 ± 0.10 ^a^	16.19 ± 1.00 ^a^
MPH	0.78 ± 0.08 ^a^	12.09 ± 0.53 ^b^
MPH_SGID	0.65 ± 0.14 ^a^	14.34 ± 0.44 ^a,d^

**Table 9:** Radical scavenging activities of FPHs before and after SGID: BWPH-A: blue whiting protein hydrolysate A; BWPH-A_SGID: blue whiting protein hydrolysate-A post SGID; BWPH-B: blue whiting protein hydrolysate B; BWPH-B_SGID: blue whiting protein hydrolysate-B post SGID; SPH: sprat protein hydrolysate; SPH_SGID: Sprat protein hydrolysate post SGID; HPH: hake protein hydrolysate; HPH_SGID: hake protein hydrolysate post SGID; MPH: mackerel protein hydrolysate; MPH_SGID: mackerel protein hydrolysate post SGID. Data represent mean values ± SD (*n* = 3). Different letters in a column indicate significantly different mean values (*p* < 0.05). The results are expressed as IC_50_ (concentration of FPHs that scavenge the radicals by 50%).

	Radical Scavenging Activity (IC_50_ mg/mL Protein)				
Hydrolysate	DPPH^•^	ABTS^•+^	·OH	O_2_•–	H_2_O_2_
BWPH_A	2.45 ± 0.17 ^f,g^	28.48 ± 1.59 ^h^	1.10 ± 0.21 ^f,j,k^	5.69 ± 0.53 ^g^	7.59 ± 1.82 ^a,b,h^
BWPH-A_SGID	6.22 ± 1.64 ^c,f^	8.63 ± 0.26 ^g^	0.84 ± 0.14 ^c,h,i,j^	10.57 ± 3.04 ^c,d,g^	13.92 ± 0.79 ^e,g^
BWPH_B	22.82 ± 1.10 ^e^	125.25 ± 7.22 ^f^	3.46 ± 0.79 ^g^	6.66 ± 0.75 ^g^	22.73 ± 1.13 ^f^
BWPH-B_SGID	5.24 ± 0.24 ^c^	5.82 ± 2.76 ^a,b,c,d,e,g^	0.48 ± 0.14 ^a,b,d^	21.15 ± 0.62 ^f^	17.18 ± 1.78 ^e^
SPH	0.73 ± 0.11 ^d^	2.76 ± 0.05 ^e^	0.49 ± 0.27 ^a,b,c,d,h^	1.75 ± 0.16 ^e^	2.22 ± 0.40 ^d^
SPH_SGID	2.63 ± 0.68 ^a,b,g^	3.03 ± 0.11 ^d^	0.89 ± 0.10 ^c,k^	11.50 ± 3.05 ^d,g^	5.57 ± 1.38 ^b,d^
HPH	5.67 ± 0.29 ^c^	11.17 ± 0.27 ^c^	0.66 ± 0.16 ^a,b,c,e^	3.18 ± 0.22 ^c^	11.07 ± 0.70 ^c,h^
HPH_SGID	7.45 ± 1.12 ^c^	9.96 ± 0.19 ^b^	0.74 ± 0.02 ^b,c,f^	15.56 ± 1.15 ^a,d^	12.66 ± 0.86 ^c,g^
MPH	1.74 ± 0.04 ^b^	4.13 ± 0.12 ^a^	0.60 ± 0.11 ^a,b,i^	2.53 ± 0.13 ^b^	3.66 ± 0.13 ^b^
MPH_SGID	2.52 ± 0.12 ^a,f^	3.93 ± 0.28 ^a^	0.62 ± 0.02 ^a,j^	24.96 ± 5.99 ^a,f^	9.01 ± 0.89 ^a^

**Table 10:** Lipid peroxidation inhibition activity of FPHs before and after SGID: BWPH-A: blue whiting protein hydrolysate A; BWPH-A_SGID: blue whiting protein hydrolysate-A post SGID; BWPH-B: blue whiting protein hydrolysate B; BWPH-B_SGID: blue whiting protein hydrolysate-B post SGID; SPH: sprat protein hydrolysate; SPH_SGID: sprat protein hydrolysate post SGID; HPH: hake protein hydrolysate; HPH_SGID: hake protein hydrolysate post SGID; MPH: mackerel protein hydrolysate; MPH_SGID: mackerel protein hydrolysate post SGID. Data represent mean values ± SD (*n* = 3). Different letters in a column indicate significantly different mean values (*p* < 0.05). The results are expressed as IC_50_ (protein concentration of FPHs that inhibit peroxidation by 50%).

Hydrolysate	Lipid Peroxidation Inhibition (IC_50_ mg/mL Protein)
BWPH_A	2.25 ± 0.27 ^c,g^
BWPH-A_SGID	8.53 ± 0.51 ^f^
BWPH_B	16.02 ± 1.47 ^e^
BWPH-B_SGID	1.66 ± 0.12 ^b^
SPH	2.38 ± 0.74 ^b,c^
SPH_SGID	5.80 ± 0.65 ^d^
HPH	2.66 ± 0.07 ^c^
HPH_SGID	1.66 ± 0.03 ^b,g^
MPH	9.18 ± 2.87 ^a,c,d,f^
MPH_SGID	11.68 ± 1.31 ^a^

## Data Availability

The data presented in this study are available on request from the corresponding author.

## Abbreviations

AA: amino acid; BWPH-A: blue whiting protein hydrolysate-A; BWPH-B: blue whiting protein hydrolysate-B; DH: degree of hydrolysis; EAA: essential amino acids; EAI: emulsion activity index; ESI: emulsion stability index; FAA: free amino acids; FC: foam capacity; FPH: fish protein hydrolysate; FS: foam stability; GI: gastrointestinal; HPH: hake protein hydrolysate; IC_50_: half maximal inhibitory concentration; MPH: mackerel protein hydrolysate; NEAA: non-essential amino acids; OHC: oil holding capacity; ROS: reactive oxygen species; RT: room temperature; SGID: simulated gastrointestinal digestion; SPH: sprat protein hydrolysate; TAA: total amino acids; WHC: water holding capacity.
